# An Ultrapotent, Ultraeconomical, Antifreeze Polypeptide

**DOI:** 10.1002/adma.202420504

**Published:** 2025-08-28

**Authors:** Thomas J. McPartlon, Charles T. Osborne, Ke Wang, Rachel E. Detwiler, Konrad Meister, Jessica R. Kramer

**Affiliations:** ^1^ Department of Molecular Pharmaceutics University of Utah Salt Lake City UT 84112 USA; ^2^ Department of Biomedical Engineering University of Utah Salt Lake City UT 84112 USA; ^3^ Department of Chemistry and Biochemistry Boise State University Boise ID 83725 USA

**Keywords:** antifreeze, cryopreservation, cryoprotectant, IRI, N‐carboxyanhydride, polypeptide

## Abstract

The growth of large ice crystals during freeze and thaw events is a challenge in diverse settings from transportation and agriculture to foods and biomedicine. Design, synthesis, and evaluation of antifreeze polypeptides that inhibit ice crystal growth at µg concentrations are reported herein. The polypeptides, composed of Ala and Glu, are prepared using economical methodology, are stable after thermal events, are biodegradable, and are nontoxic to human cells. Mirror‐image polypeptides have resisted degradation and are suitable for applications with a longevity criterion. Their α‐helical conformation plays a role in antifreeze activity, but chirality does not. In proof‐of‐concept experiments, the antifreeze polypeptides could prevent damage to model protein therapeutics during repeated freeze–thaw cycles and could be applied to prevent large ice crystals in a frozen food product. These simple, economical Ala/Glu polypeptides are promising materials for diverse antifreeze applications, particularly in biological settings.

## Graphical abstract

Simple and inexpensive polypeptides composed of random copolymers of alanine and glutamic acid are effective antifreezes for applications in foods and biomedicine. The polypeptides, inspired by natural extremophile proteins, inhibit the growth of large ice crystals and prevent damage to sensitive biologics.

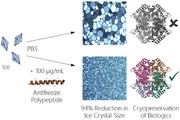

## 1 Introduction

Ice crystal formation and growth is a broad challenge affecting industries such as building materials, surface coatings in transportation, agriculture, foods, and biomedicine.^[^ ^1^, ^2^, ^3^, ^4^, ^5^ ^]^ As such, antifreezes are highly sought after to control ice growth and reduce damage caused by ice coarsening, which is the growth of large crystals at the expense of smaller crystals. In abiotic settings, glycols are widely used and effective deicing and antifreeze compounds. However, glycols are highly toxic to mammals when ingested and are also toxic to mammalian cells in culture.^[^ ^6^, ^7^ ^]^ Thus, glycols are not typically used in food technology, agriculture, or biomedicine and are environmental pollutants of concern.^[^ ^8^ ^]^

A wide variety of small molecule and macromolecular antifreeze agents have been investigated and utilized in biological settings.^[^ ^2^, ^9^, ^10^, ^11^ ^]^ In biomedicine, such as in protein and cell cryopreservation, small molecules such as sucrose, glycerol, and most commonly, dimethylsulfoxide (DMSO) are utilized. These molecules form strong hydrogen bonds with water, slowing diffusion and delaying the formation and growth of ice crystals, which causes a colligative depression of the thermodynamic freezing point and promotes vitrification rather than crystallization.^[^ ^10^ ^]^ Despite widespread use, DMSO and glycerol have well‐known toxic effects on cells and organs,^[^ ^12^, ^13^, ^14^, ^15^, ^16^, ^17^, ^18^ ^]^ which, in some cases, renders cryopreservation infeasible.

In the food industry, plant cell wall polysaccharides including carrageenan, locust bean gum, and guar gum, are common texture enhancers that prevent formation of large ice crystals.^[^ ^19^ ^]^ Unlike the small molecule solutes, polysaccharides generally do not depress the freezing point. Instead, they function via promotion and control of ice nucleation events. As a result, ice crystals have a shorter time window for growth, and thus a greater number of smaller crystals will form.^[^ ^20^ ^]^ This is a property known as ice‐recrystallization inhibition (IRI). Polysaccharides have been investigated for application in biomedicine; however, their low potency necessitates the use of very high concentrations (e.g., 200 mg mL^−1^). The requirement of such high concentrations presents a variety of challenges including osmotic stress and associated toxic effects to tissues, and excessive solution viscosity associated with processing challenges and undesirable alteration of texture in foods.^[^ ^21^ ^]^

**Figure 1:**
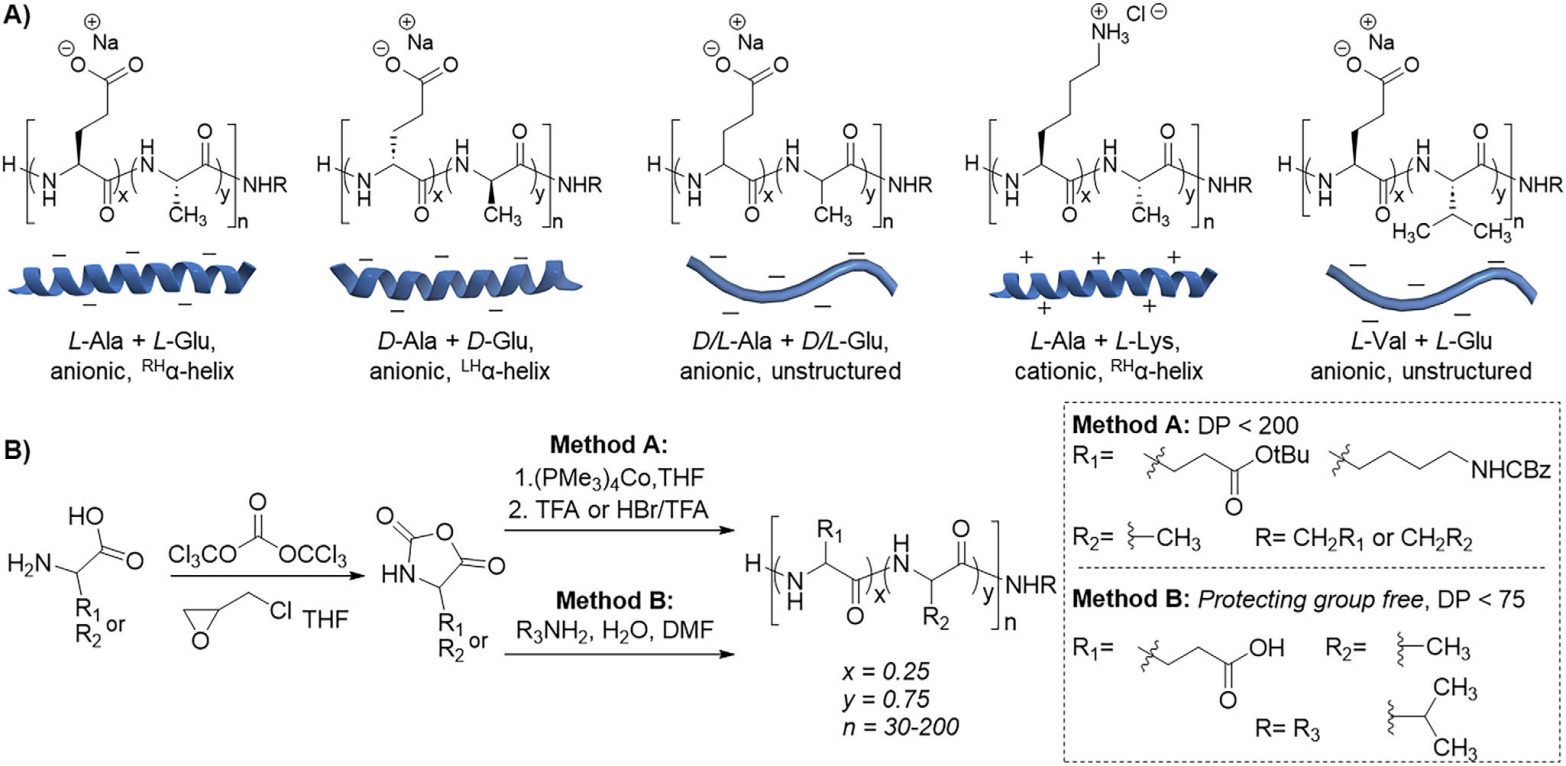
Design and synthesis of antifreeze polymer panel. A) Chemical structures, cartoon depiction, and properties of the panel. B) Conversion of amino acids into cyclic *N*‐carboxyanhydride (NCA) monomers using triphosgene, and for Glu and *t*Bu‐Glu NCAs, epichlorohydrin was added as an acid scavenger. NCA polymerization was conducted by Method A which required anhydrous/inert conditions and protecting groups but enabled preparation of 200+ residue polypeptides; or by Method B which could proceed without protecting groups in open atmosphere and aqueous condition, allowed easy tuning of the chain termini chemical group via functional amines (R_3_) but limited polypeptides to a degree of polymerization (DP) of ≈50 residues.

Inspired by the structures of natural proteins found in sub‐zero extremophiles, we designed fully synthetic polymers that efficiently inhibit ice crystal growth.^[^ ^22^, ^23^, ^24^, ^25^ ^]^ Here, we report ultrapotent, ultraeconomical, antifreeze polymers composed entirely of natural amino acids (**Figure** **1**). The polymer repeat units can be acquired for ≈$5 per kg and polymerized via scalable protecting‐group‐free methodology.^[^ ^26^, ^27^ ^]^ The polypeptides inhibit ice crystal coarsening at µg mL^−1^ concentration, are nontoxic, and are tunably biodegradable. We provide mechanistic insights toward the function of these antifreeze polymers by systematic variation of conformation, charge, and hydrophobicity, and we demonstrate proof‐of‐concept experiments for both biomedical and food industry applications.

## 2 Results and Discussion

### 2.1 Design and Synthesis of Bioinspired Antifreeze Polypeptides

Due to the toxicity and low potency (i.e 10+ wt%) of glycols, glycerol, DMSO, and polysaccharides, researchers have sought out novel, bio‐friendly antifreezes. Nature has already addressed this challenge via evolution of proteins that protect extremophile organisms from freezing damage. Antifreeze proteins and glycoproteins (AF(G)Ps) have been identified in fish, insects, plants, bacteria, and fungi adapted to survive sub‐zero temperatures.^[^ ^2^, ^22^, ^23^, ^24^, ^28^, ^29^, ^30^, ^31^, ^32^ ^]^ Despite residing in ≈56 M liquid water, AF(G)Ps bind specifically to water ordered in solid ice. Binding of the proteins to embryonic ice crystals modifies crystal shape, restricts growth, and lowers solution freezing points, thus preventing tissue damage.^[^ ^33^, ^34^, ^35^, ^36^ ^]^ Remarkably, AF(G)Ps exhibit such activities at only µg concentrations.^[^ ^1^, ^2^, ^3^, ^4^, ^5^ ^]^

AF(G)Ps evolved in a variety of sequences and structures, including globular, α‐helical, β‐helical, and glycosylated PPII (polyproline type II) helical.^[^ ^1^, ^37^ ^]^ α‐Helical type 1 antifreeze proteins (AFP1) and AFGPs are both relatively simple, consisting of alanine‐rich sequences. AFP1s are 3.3–4.5 kDa with hydrophobic residues (A, L) comprising ≈70% of the sequence with the balance being neutral/polar (T, S, N) or charged (D, E, K, R) residues.^[^ ^25^, ^38^, ^39^ ^]^ PPII helical AFGPs have a similar Ala content with the balance being almost entirely glycosylated Thr.^[^ ^35^, ^40^ ^]^ Broadly, the generally accepted mechanism of action of both is an adsorption‐inhibition model, where proteins adsorb to the ice surface and impede growth by inhibiting approach of liquid water.^[^ ^41^, ^42^, ^43^ ^]^ However, molecular details of binding events have been elusive. A variety of competing data and theories have been proposed for the roles of specific residues and the role of preordered water layers.^[^ ^33^, ^34^, ^38^, ^41^, ^44^, ^45^, ^46^, ^47^, ^48^, ^49^, ^50^, ^51^, ^52^, ^53^, ^54^ ^]^ Computational work sheds light on the potential evolutionary drivers of the conserved Ala‐content and the residues’ role in ice‐binding. These data indicated that Ala methyl groups could specifically nest into ice‐surface cavities and driven by the entropy of dehydration, provide a driving force for binding.^[^ ^55^, ^56^ ^]^ Our lab experimentally corroborated this model via observation of decreasing IRI activity with decreasing Ala content in synthetic Ala/glyco‐Thr copolypeptides.^[^ ^57^ ^]^

Despite their desirable properties, AF(G)Ps have not found broad application because harvest from extremophile organisms is expensive and laborious. Furthermore, purification challenges typically render the products as mixtures of isoforms and with potential contamination with strong allergens.^[^ ^37^ ^]^ Recombinant synthesis has been achieved but the purified proteins remain prohibitively expensive.^[^ ^58^, ^59^, ^60^ ^]^ Therefore, diverse materials have been explored as AF(G)P mimics including peptides, peptoids, polyvinylalcohol, cellulose, surfactants, quantum dots, and graphene oxide.^[^ ^2^, ^9^, ^11^, ^61^, ^62^, ^63^, ^64^, ^65^, ^66^, ^67^, ^68^, ^69^, ^70^, ^71^, ^72^, ^73^, ^74^, ^75^, ^76^, ^77^, ^78^, ^79^, ^80^, ^81^, ^82^, ^83^, ^84^, ^85^ ^]^ Yet, orders of magnitude higher concentration are required for IRI activity as compared to native AG(F)Ps and many of these materials require complex syntheses. Additionally, the use of non‐natural building blocks with poor or unknown biodegradability is undesirable for most applications. The need remains for an antifreeze material with excellent IRI activity and biocompatibility balanced with cost economy.

To fill this gap, our lab and the Jeong lab recently explored *N*‐carboxyanhydride (NCA)‐derived synthetic copolymers of Ala and glycosylated Thr, glycosylated Hyp, succinylated Thr, or Lys, all of which demonstrated some level of IRI activity.^[^ ^57^, ^86^, ^87^, ^88^, ^89^, ^90^ ^]^ However, these structures require multistep syntheses with protecting group manipulations, which is a barrier to scalability. Some also relied on postpolymerization modification chemistry with incomplete functionalization. Further, it is well known that polymers and peptides with a high density of cationic groups, such as Lys, are cytotoxic due to disruption of the cellular plasma membrane.^[^ ^91^, ^92^, ^93^ ^]^ Considering the established role of hydrophobic Ala in AF(G)Ps and these mimics, we sought to conserve this design feature while pursuing our goal of economical, biodegradable, and nontoxic antifreeze materials.

PolyAla is not water soluble and therefore not an antifreeze candidate. Hydrophilic groups are required to solubilize Ala‐rich structures and might also affect ordering of liquid water approaching the ice surface or affect inter‐ or intrachain interactions. Glu is particularly attractive as the hydrophilic component since polyGlu is biocompatible, biodegradable, nonimmunogenic, and has been extensively applied in biomedicine.^[^ ^94^ ^]^ Additionally, a recent report indicated potential for preparation of polyGlu without the need for protecting groups.^[^ ^95^ ^]^ It is notable that Glu and Ala are extremely economical building blocks with human‐consumption grade material available for bulk purchase on the public market (i.e., Amazon) for ≈$5 kg^−1^.^[^ ^26^, ^27^ ^]^ By comparison, recombinant AFPs are commercially available for $2000–$5000 mg^−1^.^[^ ^58^, ^59^, ^60^ ^]^ AFPs sourced from fish blood are less expensive at ≈$400 g^−1^, but are known to contain impurities, including potential allergens.^[^ ^37^ ^]^ NCA polymerization is an attractive alternate route to peptides/polypeptides since it is rapid, scalable, and previously validated for commercial scale.^[^ ^96^, ^97^, ^98^ ^]^

We explored two NCA polymerization methods to obtain statistical copolypeptides: traditional anhydrous polymerization with protecting groups (Method A) and protecting‐group‐free aqueous polymerization (Method B) (Figure 1B). In Method A, the cobalt initiator is sensitive to oxygen, moisture, and labile protons. Yet, this initiator is known to offer fast initiation kinetics, side reaction suppression, controlled and living polymerization, and to yield high molecular weight (MW) polymers of low dispersity.^[^ ^97^, ^99^ ^]^ The primary amine initiators utilized in Method B typically result in side reactions that limit polymer length and increase dispersity, but are potentially more tolerant to oxygen, moisture, and labile protons (i.e., free acid on Glu).^[^ ^95^, ^100^ ^]^ Though some protecting groups are readily removed, such as *tert*‐butyl (*t*Bu) removal by trifluoroacetic acid (TFA) as utilized here, avoidance of protecting groups lowers starting material cost, saves researcher time, and is greener overall. Additionally, we explored both methods so that we could access a variety of polymer chain lengths since antifreeze activity is known to increase with MW in native and synthetic AFGPs.^[^ ^57^, ^73^, ^87^, ^101^ ^]^

NCAs of *L*‐Ala (A^L^), *L*‐Glu (E^L^), and *t*Bu‐*L*‐Glu (*t*Bu‐E^L^) were prepared in ≈90% yield by treatment of the corresponding amino acids with either triphosgene or phosgene solution in anhydrous tetrahydrofuran (THF) using established procedures.^[^ ^95^, ^102^, ^103^, ^104^ ^]^ For *t*Bu‐E^L^ and E^L^ NCAs, acid scavenger epichlorohydrin was utilized in the reaction. Conveniently, NCAs were amenable to crystallization and preparation on multigram scale. We chose a ratio of 3:1 A^L^:E^L^ based on the Ala content of natural winter flounder AFP1 (wfAFP1), shorthorn sculpin AFP1 (ssAFP1), and notothenoid AFGPs. Additionally, we previously explored polypeptides with varying ratios of A^L^ to glycosylated *L*‐Thr and found that reduction to 50% A^L^ eliminated all antifreeze activity but polypeptides with >80% A^L^ were not soluble in water.^[^ ^57^ ^]^

**Figure 2:**
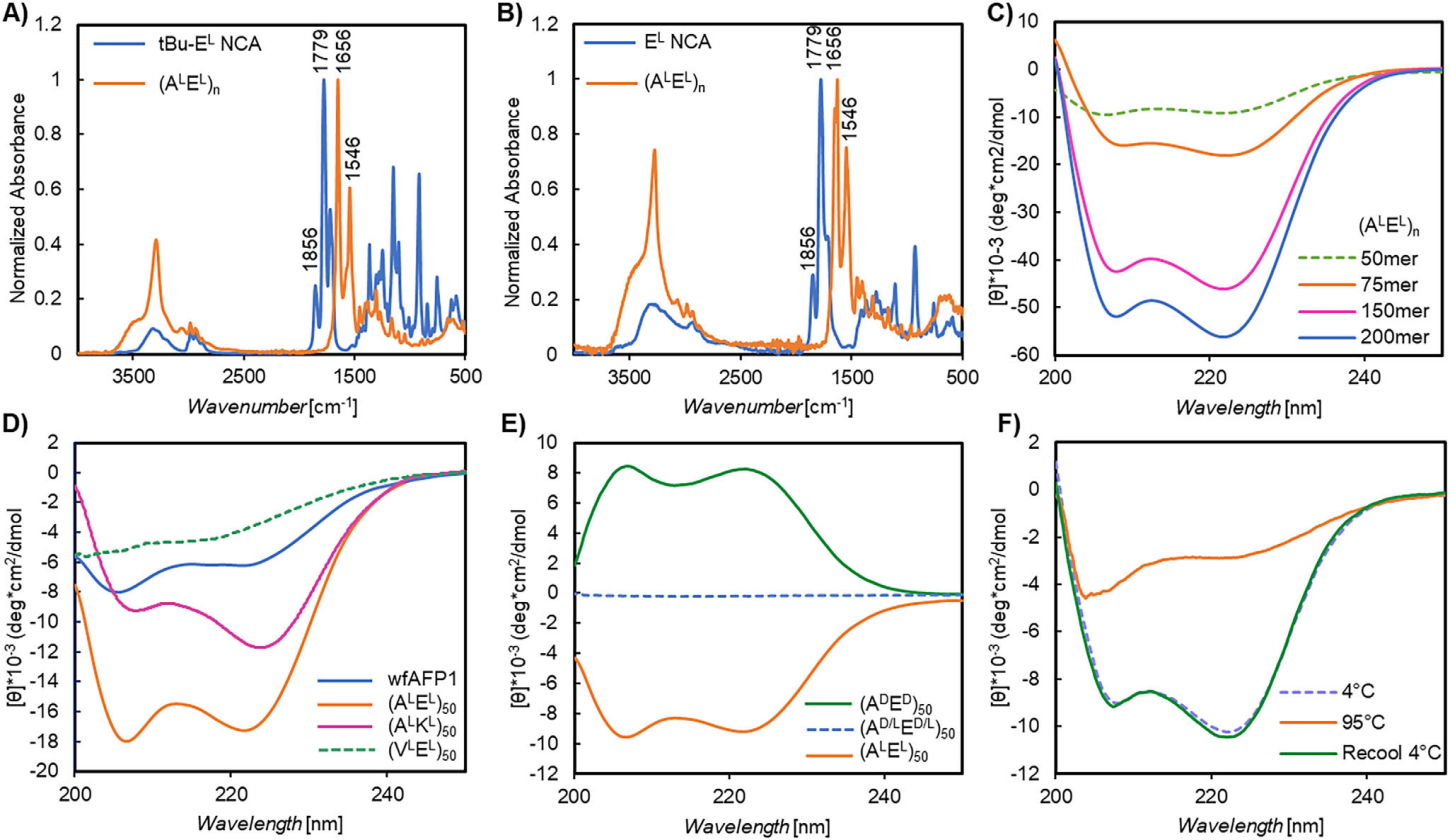
Polypeptide structural characterization. A) FTIR traces of *t*Bu‐E^L^ NCA as compared to (A^L^E^L^)_50_. B) FTIR traces of protecting‐group‐free E^L^ NCA as compared to (A^L^E^L^)_50_. C–E) CD spectra of polypeptides in PBS buffer at 20 °C. Data in C) indicates length dependent helical propensity of (A^L^E^L^)*_n_*; D) reveals that (A^L^E^L^)_50_ and (A^L^K^L^)_50_ adopt right‐handed α‐helices similar to that of winter flounder AFP1 but (V^L^E^L^)_50_ is disordered; and E) indicates that mirror‐image (A^D^E^D^)_50_ adopts a left‐handed α‐helix while racemic (A^L/D^E^L/D^)_50_ is disordered. F) CD spectra in PBS buffer (average of three scans) of (A^L^E^L^)_50_ at 4, 95, and at 4 °C after heating, demonstrating that the structure is thermally reversible and, by comparison to 20 °C data in E, that helical order increases slightly as the solution approaches freezing.

A^L^ and E^L^ or *t*Bu‐E^L^ NCAs were combined at a molar ratio of 3:1 and polymerized by Method A or Method B (vide supra). A^L^ and *t*Bu‐E^L^ NCAs were polymerized using (PMe_3_)_4_Co initiator in anhydrous THF under inert atmosphere, while A^L^ and E^L^ were polymerized using hexylamine in 1:1 water:dimethylformamide (DMF) in open air. Reaction progress was monitored by attenuated total reflectance Fourier‐transformed infrared spectroscopy (ATR‐FTIR). Reaction times were rapid (i.e., <60 min.) for both methodologies. In all cases, complete monomer consumption was evidenced by the disappearance of NCA carbonyl stretches at 1856 and 1779 cm^−1^ and emergence of the peptide carbonyl amide stretch at 1656 and amide bend at 1546 cm^−1^_._ Polymers containing *t*Bu‐E^L^ were deprotected by treatment with TFA for 30 min. All copolypeptides were converted to sodium salts by treatment with aqueous sodium bicarbonate, purified by spin filtration, and lyophilized. (A^L^_0.75_‐*stat*‐E^L^_0.25_)*_n_* copolypeptides, abbreviated hereafter as (A^L^E^L^)*_n_*, were characterized by ATR‐FTIR, ^1^H NMR, and size exclusion chromatography coupled to multiangle light scattering and refractive index (SEC/MALS/RI) in Dulbecco's phosphate buffered saline (DPBS). Materials prepared using both polymerization methods were spectroscopically identical (**Figure** **2**A,B) and had unimodal distributions (see the Supporting Information for representative polymer characterization data).

Despite the need for the *t*Bu protecting group, anhydrous solvent, and inert atmosphere, we found the cobalt initiator in Method A advantageous in that we could readily tune copolypeptide degree of polymerization (DP) to ≈200 amino acids by variation of the monomer:initiator ([M]:[I]), and polymers had superbly low dispersities (Ð= 1.11–1.16). Since IRI activity increases with MW in native AFGPs, and in our previously reported glycopolymers,^[^ ^57^, ^73^, ^87^, ^101^ ^]^ we prepared (A^L^E^L^)*_n_* from 30 to 200 residues. We did not explore higher MWs since copolypeptides with DP >200 had reduced aqueous solubility. With hexylamine initiator, we could reproducibly prepare copolypeptides of ≈50 residues and Ð= 1.30–1.40 using an [M]:[I] of 50:1. However, similar to a prior report,^[^ ^95^ ^]^ DP did not increase linearly with higher [M]:[I] ratios. We explored varying the amine initiator to benzylamine and reaction water content from 0% to 50% in DMF, but longer chains did not form. Regardless, we found Method B highly attractive since the streamlined synthesis can be conducted in open air, in mixed aqueous solvent, and using reduced cost, protecting‐group‐free building blocks. Overall, the NCA method offers a rapid, scalable, economical, and convenient route to synthesize antifreeze polymers free of potential contamination with strong allergens.

Besides chain length, we sought additional structure‐function information to optimize and rationalize activity. Therefore, we explored variation of structural elements including hydrophobic residue identity, conformation, chirality, and charge. To this end, we prepared and polymerized *D*‐Ala (A^D^) and *D*‐Glu (E^D^) NCAs to probe activity of mirror image (A^D^E^D^)_50_ or racemic (A^L/D^E^L/D^)_50_. The mirror image structure possesses identical hydrophobicity and charge as (A^L^E^L^)_50_ but should form α‐helices of opposite handedness. The racemic structure will also possess identical hydrophobicity and charge as (A^L^E^L^)_50,_ but the conformation should be disordered “random coils” since equimolar mixtures of amino acids of opposite chirality typically inhibit formation of ordered structures. To probe the effect of charge, we prepared and polymerized *N*‐ε‐carbobenzyloxy‐L‐lysine (Z‐K) NCA with A^L^ NCA, which, after removal of the Z‐group with HBr, yielded cationic (A^L^K^L^)_50_. Finally, using the previously described NCA chemistry, we altered copolypeptide hydrophobicity by substituting the A^L^ residues for *L‐*Val, *L‐*Leu, or *L‐*Gly to prepare (V^L^E^L^)_50_, (L^L^E^L^)_50_, or (G^L^E^L^)_50_. (V^L^E^L^)_50_ was soluble in water; however, we found that (L^L^E^L^)_50_ and (G^L^E^L^)_50_ had very poor aqueous solubility and therefore were not advanced as antifreeze candidates.

### 2.2 Characterization of AFP Conformation

ATR‐FTIR and circular dichroism spectroscopy (CD) were utilized to examine the secondary structures of our antifreeze candidates. Both techniques utilize the energy and intensity of light absorption by peptide bonds to reveal characteristics about bond orientation. ATR‐FTIR spectra were obtained as thin films cast from water while CD spectra were obtained in PBS. Both methodologies clearly indicate (A^L^E^L^)_n_ adopts a classic, right‐handed α‐helix (^RH^α‐helix). As shown in Figure 2A,B, amide I and II (C = O stretching and N–H bending) peak frequencies were observed between 1640–1660 and 1540–1550 cm^−1^, which are characteristic of peptides in helical or disordered conformations.^[^ ^105^, ^106^, ^107^ ^]^ Amide η→π* and π →π* transitions observed by CD also indicated a classic ^RH^α‐helical conformation (Figure 2C, amide absorptions at 208 and 222 nm).^[^ ^108^, ^109^, ^110^, ^111^ ^]^ Similar to other polypeptide materials, helical stability increased with chain length.^[^ ^112^, ^113^ ^]^

Charge appeared to have minor effects on conformation. ^RH^α‐Helices were observed for both (A^L^E^L^)_50_ and (A^L^K^L^)_50_, but the absorbance magnitudes differed (Figure 2D). Differences in peptide bond energetics could be explained by the longer Lys sidechain placing the charged group farther from the peptide backbone, as well as differences in ionic group size and hydration sphere. In any case, our (A^L^K^L^)_50_ data concur with a previous report by Jeong et al. noting α‐helical conformation of statistical copolymers of Ala/Lys.^[^ ^88^ ^]^ As a benchmark, we also examined native wfAFP1 (purchased from A/F proteins) which, as expected, exhibited ^RH^α‐helical character. By contrast, the spectra of (V^L^E^L^)_50_ was characteristic of disordered proteins (Figure 2D).^[^ ^110^, ^114^ ^]^ This aligns with prior data ranking Val as lower helical propensity than Ala.^[^ ^115^, ^116^, ^117^ ^]^ Due to the equal but opposite rotation of light, the spectrum of mirror image (A^D^E^D^)_50_ was a near perfect mirror image of that of (A^L^E^L^)_50_ indicating a left‐handed α‐helix (^LH^α‐helix) (Figure 2E). The slight difference in absorbance intensities is likely due to very minor differences in sample concentration. As expected, the spectrum of racemic (A^D/L^E^D/L^)_50_ showed no absorbance minima or maxima since the equal mixtures of D and L stereochemistry amino acids inhibit formation of secondary structures.

Many coating, food, and biomedical applications in which antifreezes could be utilized require heat processing for sterilization, annealing, or mixing. Therefore, we investigated the stability of the (A^L^E^L^)_50_^RH^α‐helix to heat denaturation. The CD spectrum of (A^L^E^L^)_50_ in PBS buffer was obtained at 4 °C. The sample was then subjected to heating at 95 °C and then recooled to 4 °C with examination by CD at each step (Figure 2F). Since the spectra before and after heating were essentially identical, we conclude that the conformation of (A^L^E^L^)_50_ is thermally reversible, and this material is therefore attractive for applications requiring a thermal processing step. By contrast, some classes of natural antifreeze proteins are known to either fully or partially denature with heat, resulting in loss of antifreeze activity.^[^ ^118^ ^]^ Indeed, we saw partial denaturation for wfAFP1 subjected to the same conditions (see the Supporting Information).

### 2.3 Antifreeze Activity Assays

In contrast to vitrification agents like DMSO and glycols, natural AF(G)Ps exert their activity by binding to specific faces of embryonic ice crystals, influencing their overall shape and size. Unique characteristics of various AF(G)Ps, as well as techniques to measure antifreeze activity, have been reported and recently reviewed.^[^ ^1^, ^2^, ^34^, ^36^, ^119^, ^120^, ^121^ ^]^ Three properties are characteristic of ice‐binding proteins: 1) IRI, where ice recrystallization via Ostwald ripening is limited, resulting in a reduction in crystal mean grain size (MGS); 2) shaping of single crystals into characteristic morphologies; and 3) thermal hysteresis (TH) effects, which involve a noncolligative freezing point depression and gap between solution freezing and melting points. We investigated our copolypeptides for these characteristic properties.

**Figure 3:**
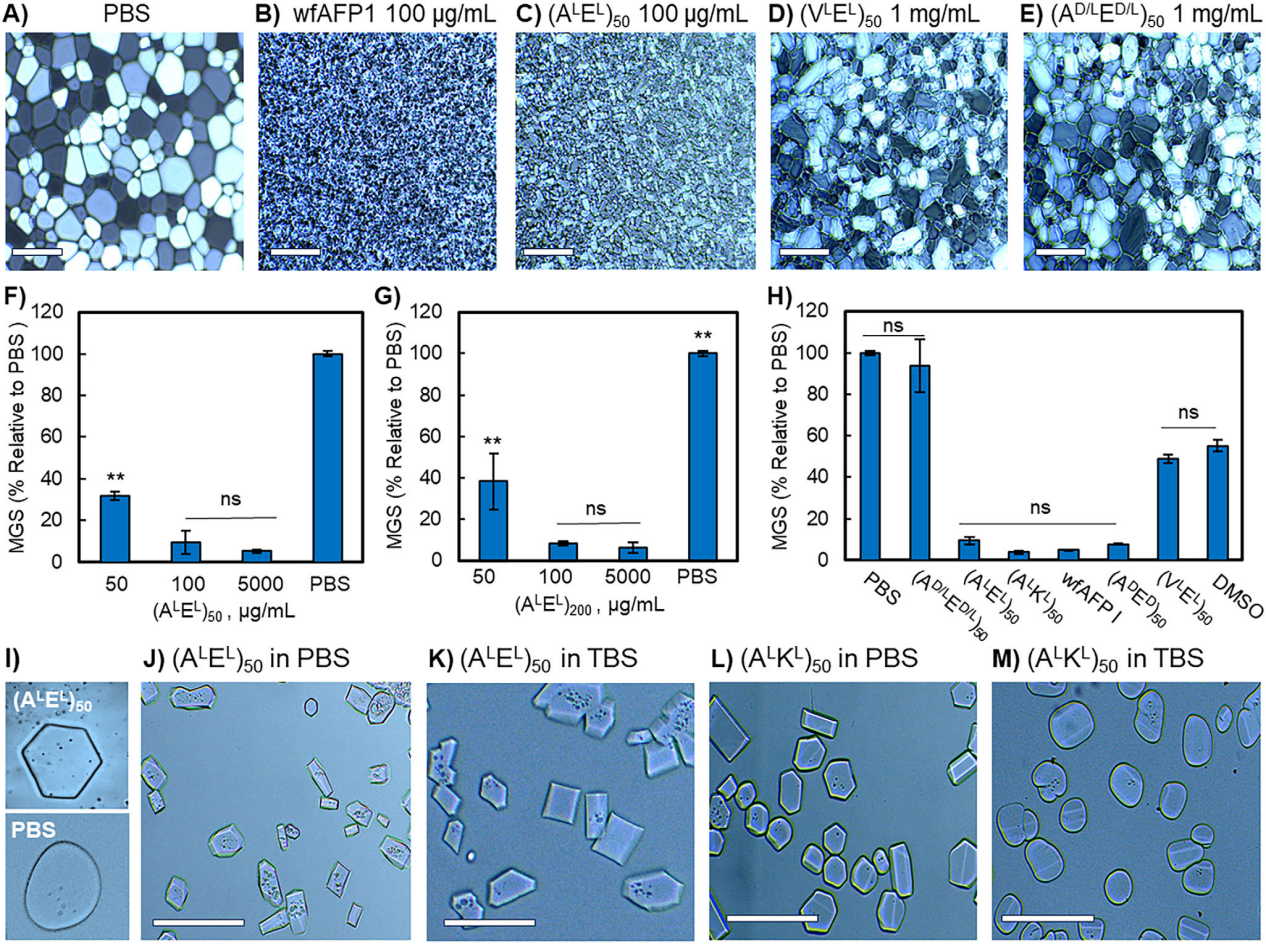
Antifreeze activity assays. A–E) Representative IRI splat assay imaging after 40 min of crystal growth for A) negative control, PBS; B) positive control, wfAFP1, 100 µg mL^−1^; C) (A^L^E^L^)_50_100 µg mL^−1^; D) (V^L^E^L^)_50_,1 mg mL^−1^; E) (A^D/L^E^D/L^)_50_, 1 mg mL^−1^. Scale bars are 200 µm. F–H) Quantified IRI assay data from a minimum of 75 crystals as % MGS relative to PBS for F) (A^L^E^L^)_50_ and G) (A^L^E^L^)_200_ at varied concentrations and H) side‐by‐side comparison of various polypeptide compositions and controls where (A^L^E^L^)_50_, (A^L^K^L^)_50_^,^ (A^D^E^D^)_50_, and wfAFP1 are 100 µg mL^−1^ in PBS, (A^D/L^E^D/L^)_50_ and (V^L^E^L^)_50_ are at 1 mg mL^−1^ in PBS, and DMSO is 100 mg mL^−1^ (10 wt%) in PBS; a minimum of three technical replicates were collected, mean and standard deviation, one‐way ANOVA with post hoc Tukey HSD tests, ** indicates *p* < 0.01. I) Dynamic ice shaping experiments comparing PBS control to (A^L^E^L^)_50_, 100 µg mL^−1^ in PBS. J–M) Dynamic ice shaping experiments comparing anionic (A^L^E^L^)_50_ to (A^L^K^L^)_50_ at 500 µg mL^−1^ in anionic PBS buffer versus cationic TBS buffer. Scale bars are 100 µm.

IRI activity assays were conducted via the cooling splat assay and observation of crystal growth over time using cryostage microscopy.^[^ ^57^, ^122^, ^123^ ^]^ Our controls were PBS, DMSO, and wfAFP1. Ice crystal MGS was quantified using image analysis software or manual sizing in cases where crystals were too small for software recognition. Data were obtained from three separate images, using 150 µm x 150 µm regions with a minimum of 75 crystals analyzed, which we previously determined to yield statistically significant representative data.^[^ ^57^ ^]^ The average and standard deviation are presented for each experiment and statistical significance was determined with a one‐way analysis of variance (ANOVA) and post hoc Tukey honestly significant difference (HSD) test. Representative IRI images are shown in **Figure** **3**A–E and additional images can be found in the Supporting Information. Quantified data are presented in Figure 3F–H.

We examined the IRI activity of (A^L^E^L^)*_n_* at varied chain lengths from 30–200 residues and at varied concentrations from 50 to 5 mg mL^−1^. Data for the 50mer and 200mer are shown in Figure 3C,F,G and data for other lengths can be found in the Supporting Information. Near total IRI was observed at concentrations of 100 µg mL^−1^ and higher for copolypeptides with 50 or more residues, and essentially no enlargement of ice crystals was observed over the 40‐min observation period. Compared to the PBS control, copolypeptides with 50+ residues reduced ice crystal MGS by 91–94% at 100 µg mL^−1^ (Figure 3F,G and the Supporting Information). By comparison, DMSO at 1000‐fold higher concentration (100 mg mL^−1^) resulted in only a 45% reduction. Lower molecular weight 30mer chains were slightly less potent, offering an 83% reduction in MGS at 100 µg mL^−1^ (see the Supporting Information). At 50 µg/mL, reduced IRI activity (60–62% MGS reduction) was observed for both the 50mer and 200mer and activity was undetectable by 10 µg mL^−1^ (see the Supporting Information).

The observed increase in IRI potency of (A^L^E^L^)*_n_* from DP 30 to 50, followed by a plateau up to DP 200, is consistent with previous reports of behavior and function of natural AF(G)Ps and analog structures. Reports by Tachibana,^[^ ^68^ ^]^ Budke,^[^ ^124^ ^]^ Sikes^[^ ^125^ ^]^ and Meister^[^ ^33^ ^]^ indicated that thermal hysteresis and ice shaping activities increase with AF(G)P chain length up to ≈15 residues, with little additional gain at higher DPs. This was shown to be due to ice adsorption kinetics, which plateau after optimal surface coverage is achieved.^[^ ^33^ ^]^ A comparable chain length threshold was observed for polyvinylalcohol, a synthetic IRI‐active polymer, where Congdon et al.^[^ ^126^ ^]^ reported an onset of activity around 20 repeat units, followed by saturation at higher DPs. These thresholds likely reflect the minimum lengths needed to stabilize a conformation that presents an organized surface for ice binding.^[^ ^33^ ^]^ Collectively, these findings by our lab and others suggest that once the minimal structural requirements for ice interaction are met, optimal surface coverage and adsorption kinetics are achieved and further increases in chain length do not significantly enhance antifreeze efficacy.^[^ ^33^ ^]^ Ultimately, we found the lack of MW‐dependence after ≈50 residues to be an advantageous result since we could use the cleaner, greener, protecting‐group‐free method to reproducibly prepare these structures.

The IRI properties of our copolypeptide panel facilitate insights into structure‐function relationships in these materials. Conformation and structural order appear to play essential roles. IRI activity was completely abrogated with the conformationally‐disordered, random coil, (A^D/L^E^D/L^)_50_, even at 10x the concentration at which (A^L^E^L^)_50_ achieves total IRI (Figure 3E–H). Racemic (A^D/L^E^D/L^)_50_ is composed of the exact chemical moieties as the highly active ^RH^α‐helical (A^L^E^L^)_50_ but cannot form an ordered helix due to the mixture of stereocenters. As expected, since water is not chiral, enantiomeric (A^D^E^D^)_50_, which adopts a mirror image ^LH^α‐helix, had essentially identical IRI activity to that of (A^L^E^L^)_50_ (Figure 3H). These results align with prior work where solid phase peptide synthesis was used to prepare enantiomeric wfAFP1, which had activity equal to that of the L‐conformer.^[^ ^127^ ^]^ There was also no statistical significance between the activity of anionic (A^L^E^L^)_50_ and cationic (A^L^K^L^)_50_, both of which adopt a ^RH^α‐helix. Finally, the Ala‐to‐Val substitution in predominantly‐disordered‐ (V^L^E^L^)_50_ resulted in a ≈50% loss of IRI activity. Presumably, differing methyl group spacing and steric size of Val versus Ala also weaken clathrate‐like interactions with ice and reduce IRI activity.

From these collective IRI data, we conclude that both cationic and anionic hydrophilic groups are tolerated, natural *L*‐stereochemistry is not required, and that the helical peptide backbone is required for optimal function. The polypeptide alpha helical structures of (A^L^E^L^)_50_ and (A^D^E^D^)*_n_* result in ordered presentation of Ala methyls and Glu ionic groups, as compared to random coil racemic (A^D/L^E^D/L^)_50,_ which had no detectable activity. Helicity would reduce structural entropy for clusters of clathrate‐like interactions with water molecules ordered on the surface of the ice lattice. We propose that following stochastic, weak, single Ala‐ice interactions, the rigid helix presents additional, favorably spaced Ala residues in close proximity, resulting in zipper‐like adsorption to the ice. Helicity may also play a role in segregation of hydrophobic and hydrophilic residues onto opposite faces of the helix. Structural organization resulting in clearly defined amphiphilicity has been demonstrated for AFGPs, ssAFP1, and proposed for various synthetic analogues.^[^ ^55^, ^128^ ^]^

Our rationale for the data shown in Figure 3A–H is supported by prior ice‐binding data of ssAFP1, and synthesized (KAAKA_7_)*_n_* mimics, as obtained via mutation screens, ice etching, and molecular dynamics and modeling.^[^ ^125^, ^129^, ^130^ ^]^ These studies indicated that binding occurs along the [$12\bar{2}$] vector on the secondary prism plane of hexagonal ice (Miller–Bravais ($\bar{2}10$) surface). It was proposed that the peptide helix, which presents 16.7–16.9 Å spacing between every 11th amino acid side chain, aligns with the 16.7 Å periodicity of oxygen atoms on this ice surface, allowing for ideal accommodation of hydrophobic side chains within the ice surface corrugation. They also deduced that at least a threefold repeat (i.e., at least a 33‐residue sequence) was required for ice binding of ssAFP1. These data correlate with our observations of increased activity of (A^L^E^L^)*_n_* from DP 30 to 50, of reduced activity for helix‐disrupted (V^L^E^L^)_50_, and with Jeong et al.’s report that conformation‐disordered statistical copolymers of *L*‐Ala/Asp do not have detectable IRI activity.^[^ ^88^ ^]^

We also conclude that, since these statistical copolypeptides are inherently composed of a randomized mixture of sequences, sequence variability is tolerated in ice binding interactions in these structures. (A^L^E^L^)*_n_* and (A^D^E^D^)*_n_* present multiple Ala groups with favorable ≈16.7 Å spacing, but the exact position along the chain varies. This idea aligns with prior reports by our lab and Jeong et al. where glycosylated and succinylated Ala‐rich polymers also had IRI‐activity but varied sequence.^[^ ^57^, ^86^, ^87^, ^89^, ^90^ ^]^ In those cases, polymers were active at mg mL^−1^ concentrations rather than µg mL^−1^ as demonstrated for (A^L^E^L^)*_n_* and (A^D^E^D^)*_n_*. We note specific sequences have been shown to play crucial roles in many natural AFP‐ice interactions, underscoring the multiple mechanisms by which proteins and other materials can bind to the different planes of ice.^[^ ^131^, ^132^ ^]^

We next explored the ability of our IRI‐active structures to shape ice crystals, which is a classic feature of ice‐binding proteins. Dynamic ice shaping experiments, which involve isolation of single crystals, were performed on a cryostage microscope and by nanoliter cryoscopy. For solutions of (A^L^E^L^)_50_ in PBS, we observed hexagonal plates (Figure 3I). While determination of the ice binding plane based solely on crystal shape is not definitive, we note hexagonal plate crystals also form in the presence of native prism plane ice binders such as Ala‐rich AFGPs and *Marinomonas primoryensis* AFP, which both bind to the primary prism plane,^[^ ^33^, ^53^, ^124^, ^133^, ^134^ ^]^ and Ala‐rich ssAFP1, which binds to the secondary prism plane.^[^ ^37^, ^129^, ^130^, ^135^ ^]^ Therefore, we presume (A^L^E^L^)_50_ is also a prism plane ice binder to the primary, secondary, or both. By contrast, ice crystals formed from PBS solution lacking antifreeze agent were round and amorphous.

Aside from interaction with ordered water molecules on the ice surface, the copolypeptide can interact with liquid water and dissolved ions. We considered that copolypeptide charged groups could form ionic bonds with charged buffer components, which might in turn affect the rate of approach of liquid water to the ice surface or affect binding of the copolypeptide to ice. Therefore, we examined ice shaping properties of anionic (A^L^E^L^)_50_ versus cationic (A^L^K^L^)_50_ in buffers of different ionic composition. We utilized PBS containing anionic phosphate groups, or tris(hydroxymethyl)aminomethane (TBS) containing cationic amine groups. A standard concentration of 0.137 m NaCl was used in all cases.

Copolypeptide‐buffer pairs of opposite charge resulted in predominantly hexagonal and bipyramidal crystal morphologies for both (A^L^E^L^)_50_ and (A^L^K^L^)_50_, (Figure 3K,L). Curiously, the like‐charge buffer‐copolypeptide pairs had differing results. Cationic (A^L^K^L^)_50_ with cationic TBS resulted in a reduction of the ice‐shaping effects observed in anionic PBS, and mainly amorphous crystals were observed (Figure 3L,M). In contrast, ice shaping abilities of anionic (A^L^E^L^)_50_ were not strongly affected by buffer. In both PBS and TBS, crystals were predominantly hexagonal and bipyramidal (Figure 3J,K). For Figure 3J–M, we selected wide view imaging to show many crystals within the same field and allow observation of trends. We note that due to slight differences in nucleation time, crystals are captured in varied stages of growth. Therefore, it is not expected that they be of uniform shape and size. Our results of buffer‐dependent effects align with previous work where AFGP activity was significantly altered by the presence of common additives used in cryopreservation media.^[^ ^136^ ^]^ Collectively, these results could be a factor in conflicting results on the utilization of AFPs and their mimics for tissue and organ cryopreservation where some studies find the additive beneficial and others detrimental.^[^ ^137^ ^]^

One possible explanation for the difference in the like‐pair buffer‐peptide charge systems could be increased stability of intermolecular interactions for (A^L^K^L^)_50_. The Lys sidechain has two additional methylene groups as compared to Glu, and thus increased side chain hydrophobicity. This in turn affects hydration sphere, intrachain packing of amino acid side chains along the helix, and potentially interhelix clustering. In TBS, (A^L^K^L^)_50_ might have stronger interchain interactions than (A^L^E^L^)_50_ in PBS, thus reducing its solution concentration and ability to bind to the prism plane of ice. We also noted differences in helical propensity of (A^L^K^L^)50 and (A^L^E^L^)_50_ (Figure 2D). Alternatively, cooperative interactions between ions, copolypeptides, and the ice surface could be at play. In any case, we conclude that (A^L^E^L^)_50_ is an attractive antifreeze agent in that it maintains its ice‐binding capability in different buffers, which increases its potential applications.

Finally, we conducted TH assays using nanoliter cryoscopy as previously described to determine the freezing and melting points of an (A^L^E^L^)_50_ solution.^[^ ^34^, ^138^ ^]^ TH activity is defined as an observed gap between solution melting and freezing points and is characteristic of certain native AF(G)Ps.^[^ ^34^, ^35^, ^121^, ^138^, ^139^ ^]^ The melting point of a 5 mg mL^−1^ (A^L^E^L^)_50_ solution in ultrapure water was obtained by cooling the sample until ice nucleation was achieved then slowly increasing the solution temperature until a single crystal remained. This was designated as the melting point. We observed that the slow cooling of a ≈15 µm ice disc slightly below the melting point resulted in faceting and transformation into a hexagon as shown in Figure 3l. Next, the temperature was cooled at a rate of 0.075 °C min^−1^ to determine the temperature at which the single ice crystal burst and grew uncontrollably. This was designated as the freezing temperature. The melting and freezing points were determined to be essentially identical at −0.037 °C, and no thermal hysteresis activity was detected. Measurements in the absence of (A^L^E^L^)_50_ resulted in the formation of circular crystals, consistent with the lack of ice‐affine molecules and in full agreement with our dynamic ice shaping experiments.

The high IRI activity of (A^L^E^L^)_50_ but lack of TH is reasonable since some plant AFPs, as well as synthetic materials like polyvinylalcohol, also have undetectable TH but robust IRI activity.^[^ ^123^, ^140^ ^]^ Ultimately, we did not consider the lack of TH activity to be a detriment since essentially all cryopreservation applications involve conditions far below the solution freezing point. While TH activity is extremely important for freeze‐avoiding organisms like polar fish residing in waters cooled to ≈−2 °C, IRI activity is the important property for damage prevention in coatings, foods, and biomedicine which are typically subjected to temperatures of −20 to −196 °C.^[^ ^6^, ^7^, ^141^ ^]^ Overall, we conclude that (A^L^E^L^)*_n_* is an attractive cryoprotectant candidate due to the combination of high IRI activity, thermal and buffer stability, and low cost of synthesis.

### 2.4 Cytocompatibility, Degradability, and Cryoprotectant Applications

Antifreezes are needed to prevent ice‐coarsening damage in diverse settings including building materials, transportation, agriculture, foods, and biomedicine.^[^ ^6^, ^7^ ^]^ Common cryoprotectants glycols and DMSO have well known toxic effects, as well as potential negative environmental impacts. Since (A^L^E^L^)*_n_* is based entirely on the natural amino acids *L*‐Glu and *L*‐Ala, we hypothesized the structure would be well tolerated by living tissues and would be biodegradable by natural proteases. To test these ideas, we characterized the cytotoxicity and in vitro biodegradation of (A^L^E^L^)*_n_*. In certain applications, long material lifetimes are needed (i.e., coatings) and biodegradation is actually undesirable. We note that mirror image *D*‐peptides are typically resistant to proteolysis^[^ ^102^ ^]^ and, therefore, (A^D^E^D^)*_n_* might be useful in scenarios where degradation would be detrimental.

**Figure 4:**
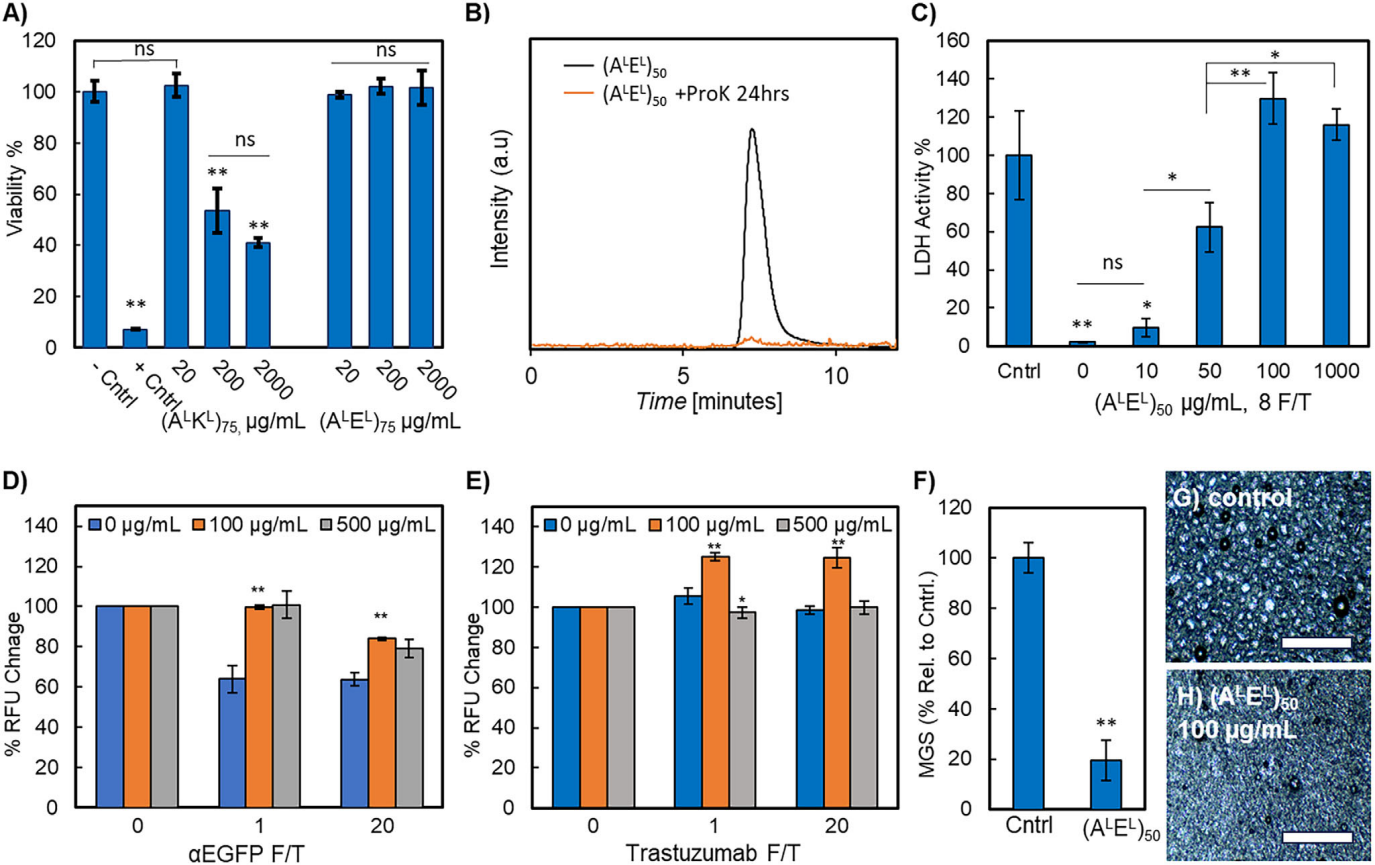
Cytocompatibility, biodegradation, and application of (A^L^E^L^)*_n_* in various freeze/thaw protection settings. A) HEK 293 cell viability as determined by CCK8 assay following 24 h incubation with anionic (A^L^E^L^)_75_ or cationic (A^L^K^L^)_75_ at the indicated concentrations; live control is media alone while dead control is Triton X‐100. B) SEC/MALS trace in DPBS of (A^L^E^L^)_50_ before and after treatment with proteinase K indicating biodegradability of (A^L^E^L^)_50_. C) Enzymatic activity of LDH after 8 F/T cycles with addition of varied concentrations of (A^L^E^L^)_50_. D) Binding activity of αEGFP after 0, 1, or 20 F/T cycles with addition of varied concentrations of (A^L^E^L^)_50_. E) Binding activity of trastuzumab after 0, 1, or 20 F/T cycles with addition of varied concentrations of (A^L^E^L^)_50_. D, E) % RFU change is the change in relative fluorescence units for the sample subjected to F/T as compared to the untreated sample that had never been frozen. F) Quantified IRI activity of 100 µg mL^−1^ (A^L^E^L^)_50_ in a frozen dairy product, as % MGS relative to the untreated control. IRI image of G) control frozen dairy product versus H) the (A^L^E^L^)_50_ supplemented sample. A,C–F) A minimum of three technical replicates were collected and a minimum of 75 crystals were measured. Standard deviation; one‐way ANOVA with post‐hoc Tukey HSD tests ** indicates *p* < 0.01.

Cytocompatibility of (A^L^E^L^)_75_ was examined in a model human cell line currently in broad laboratory use (human embryonic kidney (HEK) 293 cells). For comparison, we also quantified the cytotoxicity of cationic (A^L^K^L^)_75_ We used a commercial cell counting kit 8 (CCK8) assay, which quantifies cell proliferation and cytotoxicity. CCK8 assays were conducted after 24 h of treatment with copolypeptides at concentrations ranging from 20–2000 µg mL^−1^. We note the robustness of the experiment since the high concentration exceeds the necessary concentration for antifreeze activity by ≈40‐fold. Cells were treated with PBS as a negative control and Triton X‐100 as a positive control. At the highest concentration tested, no statistically significant effects on cellular viability were observed for anionic (A^L^E^L^)_75_ (**Figure** **4**A). In contrast, cationic (A^L^K^L^)_75_ resulted in cytotoxicity that increased with concentration and within the concentration range needed for IRI activity. These data again highlight (A^L^E^L^)*_n_* as an attractive antifreeze agent that could mitigate the toxicity concerns of current cryogenic agents.

For applications in foods and biomedicine, it is typically desirable that additives and stabilizers biodegrade via known pathways into well‐tolerated byproducts. Considering that (A^L^E^L^)_50_ is composed entirely of natural amino acids, we reasoned it would be a substrate for natural proteases. We also hypothesized that IRI‐active mirror‐image (A^D^E^D^)_50_ would be degradation‐resistant. To test this hypothesis, we utilized natural proteases proteinase K (ProK) and pepsin (Pep). ProK is a broad‐spectrum serine protease that preferentially cleaves peptide bonds adjacent to aliphatic and aromatic *L*‐amino acids,^[^ ^142^ ^]^ while Pep is an aspartic protease that has broad‐spectrum activity with a preference for aromatic or carboxylic *L*‐amino acids. Pep is found in the mammalian gastrointestinal system and is enzymatically most active at low pH.^[^ ^143^, ^144^ ^]^

Copolypeptides (A^L^E^L^)_50_ and (A^D^E^D^)_50_ were incubated with Pep or ProK for 24 h at an enzyme:substrate ratio of 1:10. Enzymes were quenched by heat treatment and samples were analyzed by SEC/MALS/RI, gel electrophoresis, or ^1^H NMR. Data for ProK with (A^L^E^L^)_50_ are shown in Figure 4B and additional data are in the Supporting Information. Disappearance of the unimodal (A^L^E^L^)_50_ SEC signal after ProK treatment clearly indicates the material was degraded by proteolysis. By contrast, the SEC signature of (A^D^E^D^)_50_ was retained after the 24 h enzyme incubation period indicating degradation resistance (see the Supporting Information). For Pep, the SEC elution time of the enzyme had considerable overlap with that of (A^L^E^L^)_50_. Therefore, we examined enzyme‐treated polymers by NMR after separation of proteolyzed fragments from intact polymer by 3 kD cutoff spin filtration. We again observed complete disappearance of signal for the *L*‐copolypeptide. This indicates (A^L^E^L^)_50_ is a substrate for Pep and should digest in the human gastrointestinal system. We note that prior to SEC and NMR, we had attempted gel electrophoresis but the low MWs of (A^L^E^L^)_50_ and (A^D^E^D^)_50_ (≈4500 Da) rendered differentiation of bands of intact versus digested material difficult. Overall, we report that *L*‐stereochemistry (A^L^E^L^)_50_ is a substrate for natural proteases and should not bioaccumulate, but that mirror‐image (A^D^E^D^)_50_ is degradation resistant and could be utilized in situations where longevity is desired.

To probe the application of (A^L^E^L^)_50_ as a cryoprotectant, we conducted proof‐of‐concept experiments in both biomedical and food applications. In biomedicine, cryogenic storage is commonly used to extend the shelf life of biological therapeutics including mRNA vaccines and protein‐based drugs such as insulin, antibodies, and enzymes. Yet, the exposure of biomolecules to freeze/thaw cycles (F/T) can result in loss of function via conformational changes, aggregate formation, adsorption to the ice‐liquid interface, and pH changes due to buffer crystallization.^[^ ^145^, ^146^, ^147^ ^]^ Major suppliers of both research‐grade and pharmaceutical‐grade antibodies and enzymes typically recommend avoiding F/T cycles.^[^ ^148^, ^149^, ^150^, ^151^ ^]^ Despite the risk, repeated F/T cycles are often needed during drug manufacturing and storage or to improve drug loading in liposomal formulations.^[^ ^152^, ^153^ ^]^ Further, accidental malfunction in temperature‐controlled systems during cold‐chain‐transport or facility storage can lead to expensive loss of material. Therefore, we explored the application of (A^L^E^L^)_50_ to cryogenic storage of protein therapeutics.

For model protein therapeutics, we selected two readily‐assayed antibodies and one enzyme for examination of function after F/T. For antibodies, we chose mouse polyclonal anti‐enhanced green fluorescent protein (αEGFP) and trastuzumab (HERCEPTIN). αEGFP, which binds to enhanced green fluorescent protein (EGFP), is an essential and widely used research laboratory tool for protein localization and visualization. Trastuzumab, which binds the human epidermal growth factor receptor 2 protein (HER2), is a lifesaving therapeutic monoclonal antibody currently in use in humans. For a model enzyme, we chose lactate dehydrogenase (LDH) since this is a common blood marker of tissue damage, is known to suffer functional loss after freezing,^[^ ^86^, ^154^ ^]^ and activity is readily assayed.

Commercially‐sourced LDH was supplied in PBS buffer and was used directly in F/T assays with or without supplementation with (A^L^E^L^)_50_. Enzymatic activity was obtained via colorimetric assay and data are normalized to the activity of untreated LDH never exposed to F/T. Samples were flash frozen, and thawed at ambient temperature. Data after 8 F/T cycles is shown in Figure 4C, while data for other cycle numbers are in the Supporting Information. The activity of LDH with no (A^L^E^L^)_50_ steadily declined with each F/T (see the Supporting Information). By 8 F/T cycles, enzymatic activity was essentially undetectable. There was no statistical difference upon addition of (A^L^E^L^)_50_ below the IRI‐active concentration (10 µg mL^−1^). However, supplementation of the LDH solution with (A^L^E^L^)_50_ at its lower limit of IRI activity, 50 µg mL^−1^, resulted in ≈60% retention of activity. We found that 100 µg mL^−1^ (A^L^E^L^)_50_ was sufficient to fully protect the enzyme from freezing damage and that activity after 8 F/T cycles was similar to enzyme that had never been frozen. We note that a prior report of a polymeric F/T stabilizer for LDH was utilized at 50‐fold higher concentration (5 mg mL^−1^) than our materials.^[^ ^86^ ^]^

To investigate antibody stability to F/T in the presence of (A^L^E^L^)_50_, we combined αEGFP or trastuzumab with increasing concentrations of copolypeptide. Commercial αEGFP was supplied in PBS buffer and used directly. Commercial pharmaceutical‐grade trastuzumab was supplied with additives α,α‐trehalose dihydrate, L‐histidine HCl monohydrate, L‐histidine, and polysorbate 20. To isolate the stabilizing effects of (A^L^E^L^)_50_, these additives were removed by spin filtration against ethylenediaminetetraacetic acid in PBS buffer. Solutions were subjected to varied F/T cycles and then assayed for retention of binding functionality. The function of both antibodies was assayed by enzyme linked immunosorbent assays (ELISAs). Antibody binding targets EFGP or HER2 were attached to copper‐coated plates via His6 sequences. Data were normalized to the binding levels of the antibody solutions prior to F/T and plotted as percentage relative fluorescence units (RFU) change.

After just one F/T cycle, antibody αEGFP suffered ≈35% loss of binding function (Figure 4D). We were pleased that the addition of 100 µg mL^−1^ (A^L^E^L^)_50_ was sufficient to fully protect the antibody from freezing damage. No further effect was observed by increasing to 500 µg mL^−1^. Surprisingly, additional F/T cycles did not further damage the function of this antibody, even up to 20 F/Ts. Similarly, trastuzumab was remarkably stable to F/T and therefore we were not able to observe cryoprotective effects of (A^L^E^L^)_50_ (Figure 4E). We were surprised by these results since the manufacturer of trastuzumab specifically recommends against F/T.^[^ ^138^ ^]^

Stability of antibodies is likely dependent upon the unique structures of origin species, and isotype. Cooling rates could also be a factor. We could find no published data for stability of αEGFP. However, due to trastuzumab's pharmaceutical status, changes in conformation and aggregation after F/T have been investigated in multiple studies.^[^ ^145^, ^155^, ^156^, ^157^ ^]^ Retention of in vitro binding function after F/T was also reported in one study.^[^ ^157^ ^]^ Minor increase in aggregation with concomitant decrease in SEC peak was observed after 3 F/T cycles, but functionality for binding was not altered. We note the binding study was conducted at 1 mg mL^−1^ antibody, but the pharmaceutically relevant formulation is 21 mg mL^−1^ and aggregation events increase with concentration. Therefore, we conducted our trastuzumab study at 21 mg/mL. As shown in Figure 4E, we observed a minor enhancement of binding in the presence of (A^L^E^L^)_50_. Since we saw a similar enhancement for LDH, we hypothesize (A^L^E^L^)_50_ could be acting as a nonspecific inhibitor of aggregate species. In any case, it is clear that (A^L^E^L^)_50_ does not interfere with enzymatic or binding activity in these cases and can be used as a simple and natural stabilizer for a variety of functional proteins.

As proof‐of‐concept for food applications, we explored the cryoprotective activity of (A^L^E^L^)_50_ in a commercial frozen dairy product. Fish AFPs have previously been investigated as ice cream additives since the formation of large ice crystals is considered undesirable in these products.^[^ ^5^ ^]^ However, the cost associated with recombinant or animal‐sourced AFPs has been limiting, and some consumers are put off by the concept of fish byproducts. (A^L^E^L^)_50_ could offer an economical, readily digestible, and animal‐free alternative. To test this, commercial ice cream was melted and supplemented with varying concentrations of (A^L^E^L^)_50_. Upon refreezing and conduction of IRI splat assays, we found substantial ice crystal growth in the untreated sample. However, for samples supplemented with (A^L^E^L^)_50_, ice crystal coarsening was strongly inhibited. Data for the lowest IRI‐active concentration is shown in Figure 4F–H, and other concentrations are in the Supporting Information. These results concur with a prior report utilizing an *L*‐Ala/Lys copolymer for IRI in ice cream,^[^ ^88^ ^]^ but in that case cytotoxicity and degradation are unknown and tenfold higher polymer concentrations were utilized as compared to our materials.

## 3 Conclusions

We report the development of ice binding polymers inspired by the structure of antifreeze proteins found in polar fish. These polymers are based entirely on the natural amino acids, alanine and glutamic acid, are nontoxic to living cells, and are digestible by natural proteases including those found in the human gastrointestinal system. The polypeptides efficiently inhibited ice crystal growth at microgram concentrations, similar to that of natural fish antifreeze proteins. Shaping of ice crystals characteristic of prism plane binding was also observed. Structural characterization indicated that helical conformation plays a role in antifreeze activity, but helical handedness does not. Mirror‐image polypeptides were equally active as antifreezes but resist protease degradation, offering an option for material longevity. The antifreeze polymers were applied in proof‐of‐concept experiments to prevent freezing damage in two classes of therapeutic proteins, antibodies and enzymes. The polymers were effective to 8+ freeze–thaw cycles and did not interfere with enzyme activity or antibody binding. Ice crystal growth could also be inhibited in a model food technology application. The antifreeze polypeptides reported here are prepared from ultracheap materials in a relatively expedient and green manner, rendering them attractive for broad applications in biomedicine, foods, agriculture, coatings and more.

## 4 Experimental Section

### Instrumentation, General Laboratory Procedures, Software, and Data Analysis

Infrared spectra were recorded and analyzed on a Bruker Alpha ATR‐FTIR Spectrophotometer using the associated Bruker software. Deionized water (18 MΩ‐cm) was obtained by passing in‐house deionized water through a Thermo Scientific MicroPure UV/UF purification unit (MilliQ). Size exclusion chromatography coupled to multiangle light scattering and refractive index detectors (SEC/MALS/RI) analyses were performed on an Agilent 1260 infinity instrument equipped with Agilent PL aquagel‐OH 30, 7.5 mm x 300 mm, 8 µm column, refractive index detector, and a miniDawn TREOS (Wyatt Technology). The copolymers were run in PBS at pH 7.4 at a rate of 1 mL min^−1^ at 25 °C. All SEC/MALS/RI samples were prepared at concentrations of 3 mg mL^−1^ and data were analyzed with Wyatt Technology Astra software. ^1^H NMR spectra were recorded on a Varian Mercury spectrometer (400 MHz) or a Bruker AVANCE NEO spectrometer (500 MHz) and are reported relative to deuterated solvent. Data were processed using MNova software. Data for ^1^H NMR are reported as follows: chemical shift (δ ppm), multiplicity, coupling constant (Hz) and integration. CD measurements of the polypeptide solutions were recorded in quartz cells with a path length of 0.1 cm, on a JASCO J‐1500 CD spectrophotometer and processed in Microsoft Excel. A Molecular Devices SpectraMax M2 microplate reader was used for our cryoprotection assays to determine fluorescence/absorbance values. A NanoDrop 2000 spectrophotometer was used to determine protein concentration. Unless otherwise specified, statistical analyses were performed using Astatsa software and one‐way ANOVA with post‐hoc Tukey HSD tests. Hexanes and dichloromethane were purified by first purging with dry nitrogen, followed by passage through columns of activated 3Å molecular sieves. THF was purified by first purging with dry nitrogen, followed by passage through columns of activated alumina. Glassware was dried at 120 °C. We used an identical recipe for preparation of PBS and DPBS which was 1.5 mmol L^−1^ KH_2_PO_4_, 137 mmol L^−1^ NaCl, 2.7 mmol L^−1^ KCl, and 8.1 mmol L^−1^ Na_2_HPO_4_ in Milli‐Q water.

### NCA Synthesis

γ‐*tert*‐butyl‐L‐glutamate NCA^[^ ^113^ ^]^ (*t*Bu‐E^L^ NCA), γ‐*tert*‐butyl‐D‐glutamate NCA^[^ ^102^ ^]^ (*t*Bu‐E^D^ NCA, L‐Glutamic acid NCA^[^ ^95^ ^]^ (E^L^), benzyloxycarbonyl‐L‐lysine NCA^[^ ^103^ ^]^ (Z‐K^L^ NCA), L‐alanine NCA^[^ ^113^ ^]^ (A^L^ NCA), and D‐alanine NCA^[^ ^102^ ^]^ (A^D^ NCA) were synthesized according to previously published methods .

### General Procedure for Statistical Copolymers Prepared by Method A Using (PMe_3_)_4_Co Catalyst

Under inert atmosphere, A^L^, A^D^, *t*Bu‐E^L^, tBu‐E^D^, or Z‐K^L^ NCAs were dissolved in anhydrous THF at concentrations of 50 mg mL^−1^. NCAs were mixed at the desired ratio of 3:1 A^L^ or A^D^ to *t*Bu‐E^L^, tBu‐E^D^, or Z‐K^L^. The (PMe_3_)_4_Co catalyst was added in one shot via syringe at the desired monomer to initiator ratio. Aliquots were removed for analysis by ATR‐FTIR. Polymerizations were complete in 1 and 3 h for 50 and 100mer, respectively, and overnight for 150mer and 200mer.

General procedure for statistical copolymers prepared by Method B using protecting‐group‐free conditions and hexylamine initiator: A^L^ and E^L^ NCAs were dissolved in DMF at 250 mg mL^−1^. Hexylamine solution at 0.077 m in DMF was added at the desired M:I ratio, followed immediately by the addition of water to yield a 1:1 DMF:water solution. The final concentration of NCA was 90 mg/mL. Aliquots were removed for analysis by ATR‐FTIR. Polymerizations were generally complete within 30 min. The polymerization solution was diluted with saturated NaHCO_3_ and dialyzed against MilliQ water using a 3 kDa MWCO membrane. Insoluble solids were removed by centrifugation and the yield of aqueous soluble material was ≈40%.

### General Method for tBu Protecting Group Removal to Yield (A^L^E^L^)*_n_*

(A^L^ *t*Bu‐E^L^)_n_ copolypeptides were dissolved in TFA at a concentration of 10 mg mL^−1^ and stirred at ambient temperature for 1 h. TFA was removed by rotary evaporation and polymers were dissolved in saturated NaHCO_3_ at a concentration of 10 mg mL^−1^. Samples were dialyzed for 3 d against MilliQ water using a 1 kDa molecular weight cutoff (MWCO) membrane. The resulting product was a white solid (60–99% yield) depending on polymer length. Polymers with 100+ residues had ≈20% insoluble portion which was removed by centrifugation prior to further experimental work.

### General Method for Z Protecting Group Removal to Yield (A^L^K^L^)*_n_*

(A^L^Z‐K^L^)*_n_* copolypeptides were dissolved in TFA and 5 eq. of 33% HBr/acetic acid was added. The reaction was stirred for 2 h. Polymers were precipitated into ether and the liquid was decanted. Solids were dissolved in water and dialyzed for 3 d against MilliQ water using a 1 kDa MWCO membrane. The resulting product was a white solid (85–99% yield). Polymers with 100+ residues had ≈20% insoluble portion which was removed by centrifugation prior to further experimental work.

### Circular Dichroism Spectroscopy

Polymers were either dissolved in PBS or Milli‐Q water. A wavelength of 214 nm, extinction coefficient of 2200 cm^−1^M^−1^and Beer's law were used to determine peptide concentration and normalize CD data. Samples were prepared at concentrations 500 or 100 µg mL^−1^. All spectra were recorded as an average of three scans. Molar ellipticity ([*θ*]) was calculated using the equation [*θ*] = (*θ*)/(10*c*l), where *θ* is measured ellipticity (mdeg), *c* is concentration (M), and *l* is path length of the cuvette (cm). For plotting, molar ellipticity was represented as [*θ*]*10^−3^.

### Dynamic Ice Shaping

To observe dynamic ice shaping 10 µL of solution containing polypeptide in 1X PBS was placed on a microscope slide and sandwiched between a cover slip. The stage was rapidly cooled at a rate of 10 °C min^−1^ to −30 °C to freeze the polymer solution. The stage was then slowly warmed to −2.5 °C at a rate of 8 °C min^−1^. Then the stage was warmed to −1.8 °C at a rate of 0.5 °C min^−1^. The stage temperature was then increased at a rate of 0.05 °C min^−1^ to −1.5 to −1 °C depending on the polypeptide solution to isolate individual crystals. The stage was then cooled at 0.02 °C min^−1^ to −2 to −1.5 °C to observe dynamic ice shaping. The stage was then toggled between melting and freezing rates to observe the ice crystal change as the temperature increased and then decreased. Images of single crystals were taken as the temperature decreased to observe ice crystal growth.

### Ice Recrystallization Inhibition Assays

Using a micropipette, 10 µL aliquots of copolypeptide solutions in PBS were dropped from 2 m through a polyvinyl chloride pipe onto a precooled slide. The slide was cooled on an aluminum block resting in a bed of dry ice. The slide containing the ice splat was quickly moved to the temperature‐controlled stage (Linkam LTS120, WCP, and T96 controller) precooled to ^−1^6.4 °C. The stage chamber was purged with N_2_ to prevent condensation from growing on the ice splat. The ice splat was annealed for 40 min and images of the ice crystals were recorded at 0, 20, and 40 min using cross polarizers (MOTICAM S3, MOTIC BA310E LED Trinocular) to observe ice recrystallization inhibition.

### Ice Recrystallization Inhibition Assay with Frozen Dairy Product

Commercially available vanilla ice cream (Tillamook brand) was allowed to fully melt at ambient temperature. Aliquot were added directly to lyophilized copolypeptide to prepare solutions containing 0–1000 µg mL^−1^ (A^L^E^L^)_50_. Cooling splat assays were conducted using 4 µL aliquots, which were pipetted onto glass slides and placed on a cryostage. The glass slide was cooled to −30 °C at a cooling rate of 30 °C min^−1^ and equilibrated for 15 min. Then, the temperature was increased to −6 °C at a heating rate of 1 °C min^−1^. The ice splat was annealed for 40 m and images were acquired.

### Quantification of Ice Recrystallization Inhibition Activity

For all polypeptide solutions, Image J (Fiji) was used to analyze the MGS of the ice crystals. For each cooling splat, three images were taken of different areas of the crystal. Briefly, ice crystals were traced by hand to determine the MGS. In the prior work, statistical analysis showed that the 150 µm x 150 µm region of the image resulted in an MGS that is representative of the entire population.^[^ ^57^ ^]^ The region of the image to analyze was randomly selected. In all cases, three images were collected for each splat assay, and the ice crystal MGS in the areas for each image were averaged. The average and standard deviations of the three images are presented for each splat.

### Thermal Hysteresis Assay

Assays were conducted 5 mg mL^−1^ (A^L^E^L^)_50_ in MilliQ water as previously described^[^ ^34^ ^]^ using a Clifton nanoliter osmometer with temperature stability of ±0.002 °C. A copper disc (1 mm thick) with seven holes (500 µm in diameter) was placed on the viewing hole of the copper plate after a droplet of immersion oil was gently placed on its bottom side. Nitrogen gas was purged into the osmometer to prevent condensation, and a humidity‐absorbing material (W. A. Hammond DRIERITE, Xenia, OH), which was sandwiched between two coverslips and sealed with hot glue, was placed on the external cover of the viewing hole. The osmometer was mounted on an upright microscope (E200, Nikon, Tokyo, Japan) and was connected to a temperature controller (model 3150/3040, Newport, Irvine, CA), which was governed by a LabVIEW program and included video output using a complementary metal‐oxide‐semiconductor camera (DMK‐33UX249, The Imaging Source, Charlotte, NC). Nanoliter volumes of the polymer solution in PBS were injected into the immersion oil using pulled glass capillaries and a syringe. The sample was then cooled until nucleation was achieved. The temperature was slowly increased to just below the melting point of the sample until a single crystal was obtained. After the melting point was documented, the temperature was decreased at a fixed rate of 0.075 °C min^−1^ and without annealing. The temperature at which the crystal “burst” (grew uncontrollably) was obtained and designated as the freezing temperature. TH activity is the difference between the melting point and the freezing (or burst) point. Measurements were performed two to four times on independent samples

### Cell Viability Assays

HEK 293 cells were plated at a density of 10000 cells per well in a 96 well plate. The cells were incubated at 37 °C in 5% CO_2_ for 24 h to allow the cells to adhere to the plate. Cells were then treated with polymer dissolved in complete media Dulbecco's Modified Eagle Medium with 10% fetal bovine serum supplemented with 1% penicillin‐streptomycin and 1% L‐glutamine) for a final polymer concentration of 0.02, 0.2, or 2.0 mg mL^−1^. Additionally, other wells of cells were treated with 100‐X Triton to kill cells as a positive control or media to serve as a negative control. The treated cells were again incubated at 37 °C in 5% CO_2_ for 24 h. The cells were then dosed with 10 µL of CCK‐8 solution (Dojindo) and incubated at 37 °C in 5% CO_2_ for 3 h. The absorbance at 450 nm of the treated cells was measured using a BioTek Synergy HTK multimode reader.

### Protease Degradation Assay

Protease K (ProK) was purchased from ThermoFisher (Cat# AM2542). The Pepsin (Pep) used was manufactured by Roche. Digestions with ProK were performed in 1x PBS pH 7.4 buffer. Digestions with Pep were done in milliQ water. The pH of the MilliQ water was adjusted to 2 using 0.1 m HCl. All reactions were run at 3 mg mL^−1^ (A^L^E^L^)_50_ or (A^D^E^D^)_50_, 10:1 substrate:enzyme ratio, at 37 °C for 24 h. Enzymes were denatured by heating the sample at 95 °C for 10 min. ProK‐treated solutions were filtered using a 0.22 µm polyethersulfone filter and subjected to SEC/MALS/RI analysis in DPBS. The total polymer mass injected was 300 µg. Due to overlap in the Pep and polymer elution in SEC, Pep‐treated samples were spin‐filtered against a 3000 MWCO membrane filter to separate digested versus intact polymer. The remaining solution in the spin filter was lyophilized and ^1^H NMR was obtained in D_2_O.

### Cryoprotection Assays with LDH

Type II LDH from rabbit muscle was acquired from Sigma Aldrich as an ammonium sulfate solution. Solutions of (A^L^E^L^)_50_ and LDH were prepared with a final concentration of LDH at 5.0 units mL^−1^ and (A^L^E^L^)_50_ at 0–1000 µg mL^−1^. Freeze–thaw cycles were conducted using 200 µL samples which were placed on dry ice for 15 min to freeze, and thawed at 25 °C in a water bath. LDH activity assays were conducted based on a literature protocol.^[^ ^86^ ^]^ Assays were completed in triplicate on 5 µL aliquots in a 96‐well plate. A 200 µL solution of 63 mM nicotinamide adenine dinucleotide + hydrogen was prepared in PBS and a 500 µL solution of 10 mm sodium pyruvate was prepared in PBS. These solutions were added to 50 mL of PBS^6^. 195 µL of this solution was added into each well, followed by the addition of 5 µL aliquots of freeze‐thawed sample, or our untreated control samples. Absorbance values at 340 nm were measured at 0 min and 20 min. LDH activity % was calculated according to the following equation, where A_0_ and A_20_ represent absorbance values at 0 and 20 min, respectively and where freeze–thawed samples were compared to the untreated control:

(1) $$\mathrm{LDH}\mathrm{activity}\left(\%\right)={\left(A_{20}-A_{0}\right)}_{\mathrm{sample}}/{\left(A_{20}-A_{0}\right)}_{\mathrm{untreated}}*100$$

### Cryoprotection Assays with Antienhanced αEGFP

EGFP (contains His6 sequence, Cat. No.: 000033P) was purchased from Applied Biological Materials. Nonfat dry milk (Cat. No.: M0841) was purchased from LabScientific. For the primary antibody, α‐EGFP (rabbit polyclonal) in PBS (Cat. No.: 16118‐T16) was purchased from SinoBiological. For the secondary antibody, α‐rabbit‐IgG‐H&L antibody (goat polyclonal, horeradish peroxidase conjugated) (Cat. No.: ab205718) was purchased from Abcam. QuantaBlu Fluorogenic Peroxidase Substrate Kit (Cat. No.: 15169) and Pierce Copper Coated High‐Capacity Plates (Cat. No.: 15148) were purchased from ThermoFisher Scientific. (A^L^E^L^)_50_ solutions were prepared in PBS at twice the final working concentration and was mixed with α‐EGFP in a 1:1 ratio. Freeze–thaw cycles were conducted using 200 µL samples which were placed on dry ice for 10 min to freeze and thawed at ambient temperature for 10 min. Solutions were stored at 4 °C before use in the ELISA.

EGFP was diluted to 250 ng mL^−1^ in PBS and immobilized onto copper‐coated plates in 100 µL aliquots. The plate was incubated on a rocker at room temperature for 1 h. The coating solution was removed, and each well was washed three times with 200 µL 0.05% PBS (prepared as previously described)13643 with the addition of 0.5 mL of Tween 20 per 1L of PBS. (All following incubation and wash steps are performed in this manner if not otherwise specified.) Unoccupied binding sites were blocked with 5% milk in PBS with 15 min incubation. The blocking solution was removed and each well was washed. The EGFP was detected with the primary α‐EGFP antibody diluted in PBS at 1:5000 with the dilution ratio relative to antibody (not to the mixture of antibody and polymer) with 1 h incubation. The primary antibody solution was removed and each well was washed. The primary antibody was detected with the secondary antibody diluted 1:1000 in PBS with 1 h incubation. The secondary antibody solution was removed and each well was washed. The secondary antibody was detected with 100 µL QuantaBlu working solution. RFU were acquired with on a Molecular Devices SpectraMax M2 microplate reader at 325 nm excitation wavelength and 420 nm emission wavelength. The data were plotted in Microsoft Excel.

### Cryoprotection Assays with Trastuzumab (Antihuman Epidermal Growth Factor Receptor 2, (HER2))

HER2 (contains His6 sequence, Cat. No.: HE2‐H5225‐100 µg) was purchased from Acro Biosystems. For the primary antibody, human pharmaceutical grade trastuzumab (HERCEPTIN, Roche) was commercially sourced from the University of Utah Pharmacy. For the secondary antibody, α‐Human‐IgG antibody (peroxidase conjugated, goat polyclonal) (Cat. No.: 609–1302) was purchased from Rockland. Trastuzumab was exchanged into 1 mM EDTA in PBS with Amicon Ultra 30 kDa MWCO spin filters (Merck Millipore, Cat. No.: UFC203024) and concentration was determined by absorbance at 280 nm on a NanoDrop 2000 spectrophotometer (147000 Da, 225 000 M^−1^cm^−1^). (A^L^E^L^)_50_ solution in PBS with 1 mM EDTA was combined with trastuzumab solution to yield a final antibody concentration of 21 mg mL^−1^ and (A^L^E^L^)_50_ 0–500 µg mL^−1^. Freeze–thaw cycles were conducted using 200 µL samples which were placed on dry ice for 10 min to freeze, and thawed at ambient temperature for 10 min. Solutions were stored at 4 °C before use in the ELISA.

HER2 was diluted to 2 µg mL^−1^ in PBS and immobilized onto copper‐coated plates in 100 µL aliquots. The plate was incubated on a rocker at room temperature for 2 h. The coating solution was removed, and each well was washed three times. Unoccupied binding sites were blocked with 5% milk in PBS with 15 min incubation. The blocking solution was removed and each well was washed. HER2 was detected with trastuzumab diluted in PBS to 1 mg mL^−1^ with 1 h incubation. The trastuzumab solution was removed and each well was washed. Trastuzumab was detected with the secondary antibody diluted 1:20 000 in PBS with 1 h incubation. The secondary antibody solution was removed and each well was washed. The secondary antibody was detected with 100 µL QuantaBlu working solution. RFU were acquired with a Molecular Devices SpectraMax M2 microplate reader at 325 nm excitation wavelength and 420 nm emission wavelength. The data were plotted in Microsoft Excel.

### Statistical Analysis

For all CD, IRI, cytotoxicity, and enzyme and antibody F/T assays a minimum of three technical replicates were collected and for IRI, a minimum of 75 crystals were measured. Averages and standard deviations were calculated using Microsoft Excel software. IRI, cytotoxicity, and F/T data were analyzed with one‐way ANOVA with post‐hoc Tukey HSD tests using Stata software, as noted in figure captions.

## Conflict of Interest

The authors declare no conflict of interest.

## Supporting information



Supporting Information

## Data Availability

The data that support the findings of this study are available from the corresponding author upon reasonable request.
