# Supplementary material for: An Ultrapotent, Ultraeconomical, Antifreeze Polypeptide
Source: Adv Mater. 2025 Aug 28;38(4):e20504. doi: 10.1002/adma.202420504 (PMC12810662; doi:10.1002/adma.202420504)
Supplement: Supplementary file 1 — Supporting Information [file ADMA-38-e20504-s001.pdf]

ADVANCED MATERIALS

Supporting Information

for *Adv. Mater.*, DOI 10.1002/adma.202420504

An Ultrapotent, Ultraeconomical, Antifreeze Polypeptide

*Thomas J. McPartlon, Charles T. Osborne, Ke Wang, Rachel E. Detwiler, Konrad Meister and Jessica R. Kramer**

Supporting Information

An Ultra-Potent, Ultra-Economical, Antifreeze Polypeptide

*Thomas J. McPartlon[1], Charles T. Osborne[2], Ke Wang[1], Rachel E. Detwiler[2], Konrad Meister[3], Jessica R. Kramer[1,2]**

1. Department of Molecular Pharmaceutics, University of Utah. 2. Department of Biomedical Engineering, University of Utah. 3. Department of Chemistry and Biochemistry, Boise State University, Boise, ID 83725.

E-mail: jessica.kramer@utah.edu

Table of Contents

I. Synthetic Procedures 1
II. Supplementary Tables and Figures 3
III. NMR Spectra 14
IV. ATR-FTIR 17

I. Synthetic Procedures

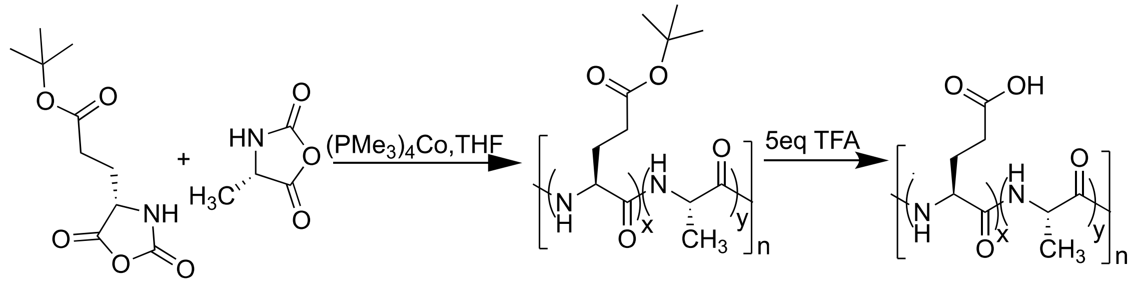

Scheme S1. Copolymerization of *t*Bu-E^L and A^L NCAs using Method A, followed by removal of tBu protecting groups to yield $(A^LE^L)_n$. Identical procedures were used to prepare $(A^{L/D}E^{L/D})_n$ and $(A^DE^D)_n$ (not pictured).

General procedure for statistical copolymers prepared by Method A using $(PMe_3)_4Co$ catalyst. Under inert atmosphere, A^L, A^D, *t*Bu-E^L, *t*Bu-E^D, or Z-K^L NCAs were dissolved in anhydrous THF at concentrations of 50 mg/ml. NCAs were mixed at the desired ratio of 3:1 A^L or A^D to *t*Bu-E^L, *t*Bu-E^D, or Z-K^L. The $(PMe_3)_4Co$ catalyst was added in one shot via syringe at the desired monomer to initiator ratio. Aliquots

were removed for analysis by ATR-FTIR. Polymerizations were complete in 1 and 3 hours for 50 and 100mer, respectively, and overnight for 150mer and 200mer.

General procedure for statistical copolymers prepared by Method B using protecting-group-free conditions and hexylamine initiator. A^L and E^L NCAs were dissolved in DMF at 250 mg/mL. Hexylamine solution at 0.077M in DMF was added at the desired M:I ratio, followed immediately by the addition of water to yield a 1:1 DMF:water solution. The final concentration of NCA was 90 mg/mL. Aliquots were removed for analysis by ATR-FTIR. Polymerizations were generally complete within 30 minutes. We also explored 0, 5 and 15% water in DMF with NCA concentrations 90–200 mg/mL at an M:I ratio of 50:1. However polymerization completion times increased to 2–8 hours. After complete monomer consumption, the polymerization solution was diluted with saturated $NaHCO_3$ and dialyzed against MilliQ water using a 3kDa MWCO membrane. Insoluble solids were removed by centrifugation and the yield of aqueous soluble material was ca. 40%.

General method for *t*Bu protecting group removal to yield (A^LE^L)$_n$. (A^L*t*Bu-E^L)$_n$ copolypeptides were dissolved in TFA at a concentration of 10 mg/mL and stirred at ambient temperature for 1 hour. TFA was removed by rotary evaporation and polymers were dissolved in saturated $NaHCO_3$ at a concentration of 10 mg/mL. Samples were dialyzed for 3 days against MilliQ water using a 1kDa MWCO membrane. The resulting product was a white solid (60–99% yield) depending on polymer length. Polymers with 100+ residues had ~20% insoluble portion which was removed by centrifugation prior to further experimental work.

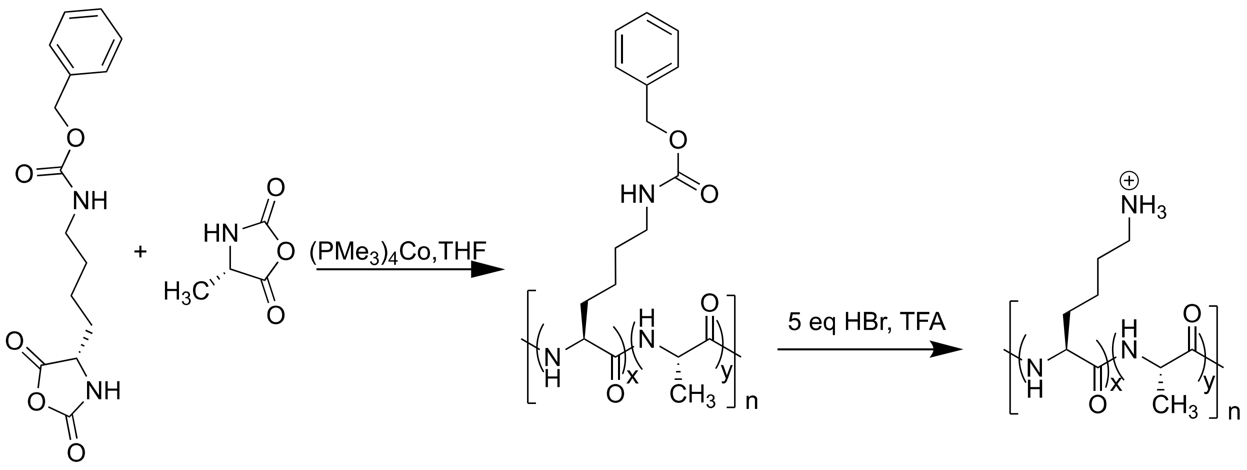

Scheme S2. Copolymerization of Z-K^L and A^L NCAs using Method A, followed by removal of Z protecting groups to yield (A^{L}K^L)$_n$.

General method for Z protecting group removal to yield (A^{L}K^L)$_n$. (A^{L}Z-K^L)$_n$ copolypeptides were dissolved in TFA and 5 eq. of 33% HBr/acetic acid was added. The reaction was stirred for 2 hours. Polymers were precipitated into ether and the liquid was decanted. Solids were dissolved in water and dialyzed for 3 days against MilliQ water using a 1kDa MWCO membrane. The resulting product was a white solid (85–99% yield). Polymers with 100+ residues had ~20% insoluble portion which was removed by centrifugation prior to further experimental work

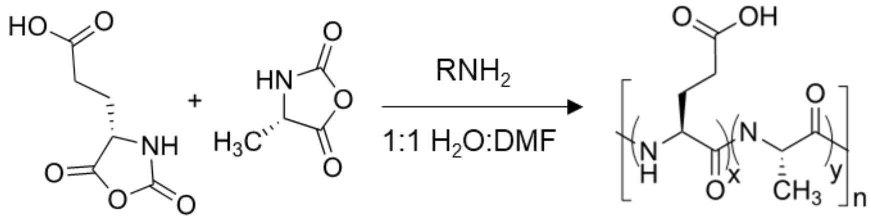

Scheme S3. Copolymerization of E^L and A^L NCAs using Method B to yield (A^{L}E^L)$_n$.

II. Supplementary Tables and Figures

Polypeptide	Abbrev.	M_n[a]	M_n[b]	DP[c]	Đ[d]	Initiator
(L-Glu$_{0.25}$-*stat*-L-Ala$_{0.75}$)$_{50}$	(A^{L}E^L)$_{50}$	4,635	4,498	48	1.11	$(PMe_3)_4Co$[e]
(L-Glu$_{0.25}$-*stat*-L-Ala$_{0.75}$)$_{100}$	(A^{L}E^L)$_{100}$	8,910	6,524	72	1.16	$(PMe_3)_4Co$[e]
(L-Glu$_{0.25}$-*stat*-L-Ala$_{0.75}$)$_{150}$	(A^{L}E^L)$_{150}$	13,185	13,180	150	1.14	$(PMe_3)_4Co$[e]
(L-Glu$_{0.25}$-*stat*-L-Ala$_{0.75}$)$_{50}$	(A^{L}E^L)$_{50}$	`4,376	4,155	45	1.34	Hexylamine[f]

Table S1: Representative analytical data for preparation of Ala-rich statistical copolypeptides. [a] Theoretical number average molecular weight, M_n. [b] Observed M_n as determined by SEC/MALS/RI in DPBS [c] Observed degree of polymerization, DP. [d] Polymer dispersity, Đ, as determined by SEC/MALS/RI. [e] $(PMe_3)_4Co$[e] initiator reactions were conducted using tBu-E^L and A^L in anhydrous THF under N_2, ambient temperature. [f] Hexylamine initiator reactions were conducted using E^L and A^L in 1:1 DMF:water in open atmosphere and ambient temperature.

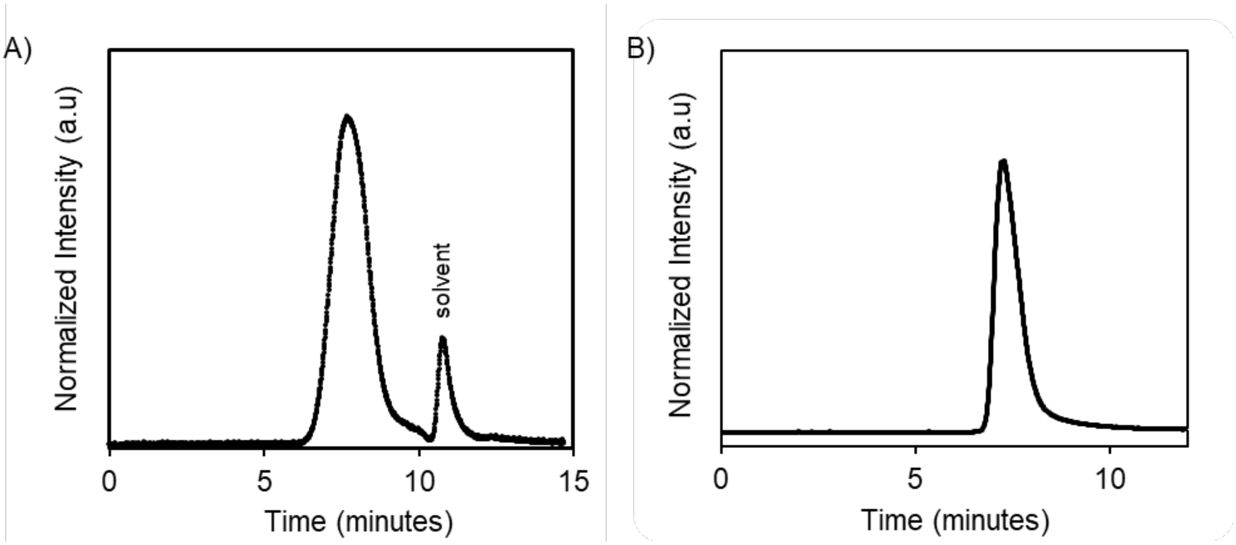

Figure S1. Representative SEC/MALS traces DPBS indicating unimodal distribution of $(A^LE^L)_{50}$ made by A) polymerization method A and B) polymerization method B.

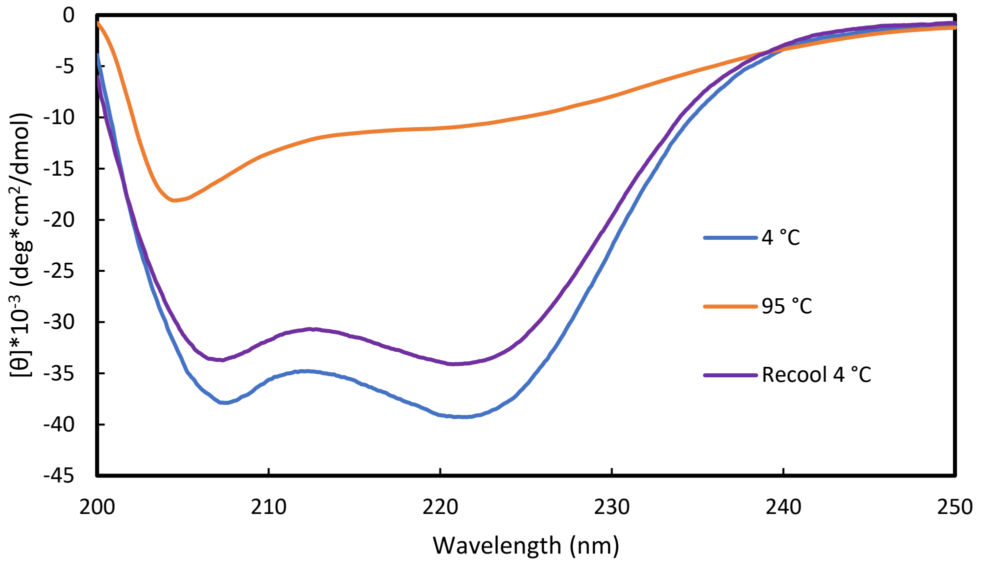

Figure S2. Variable temperature CD spectra of wfAFP1 in PBS at 500 μg/mL. The sample was exposed to the following temperature series: 4 °C → 95 °C → 4 °C.

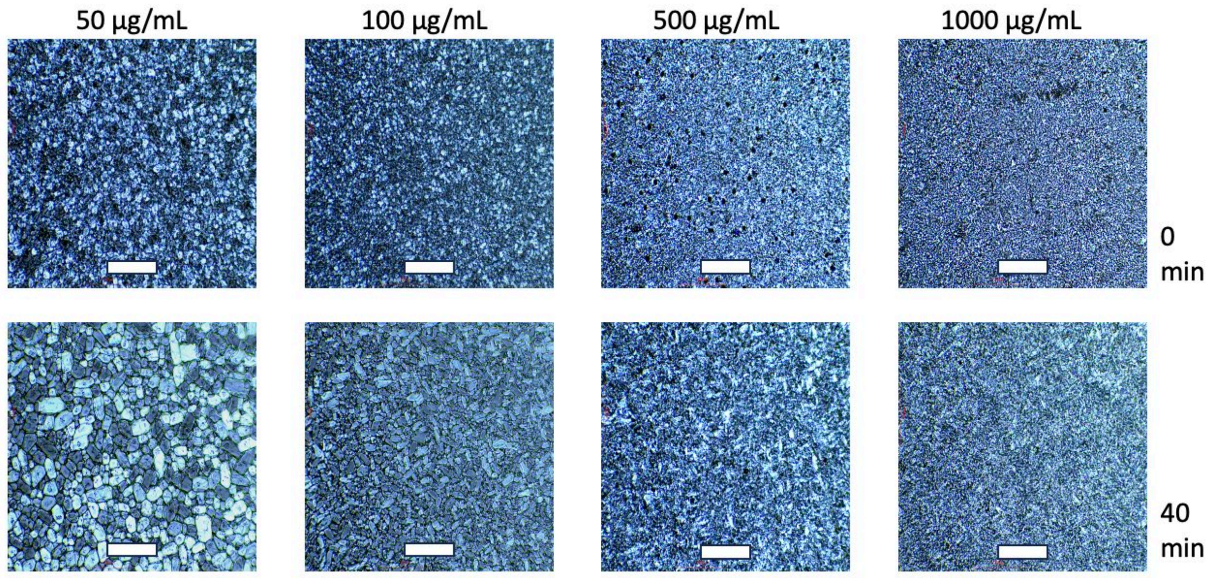

Figure S3. Ice recrystallization inhibition assay for Method A, cobalt complex-initiated, $(A^LE^L)_{50}$ dissolved at varying concentrations in PBS, at 0 minutes and 40 minutes of crystal growth. Scale bar is 200 μm.

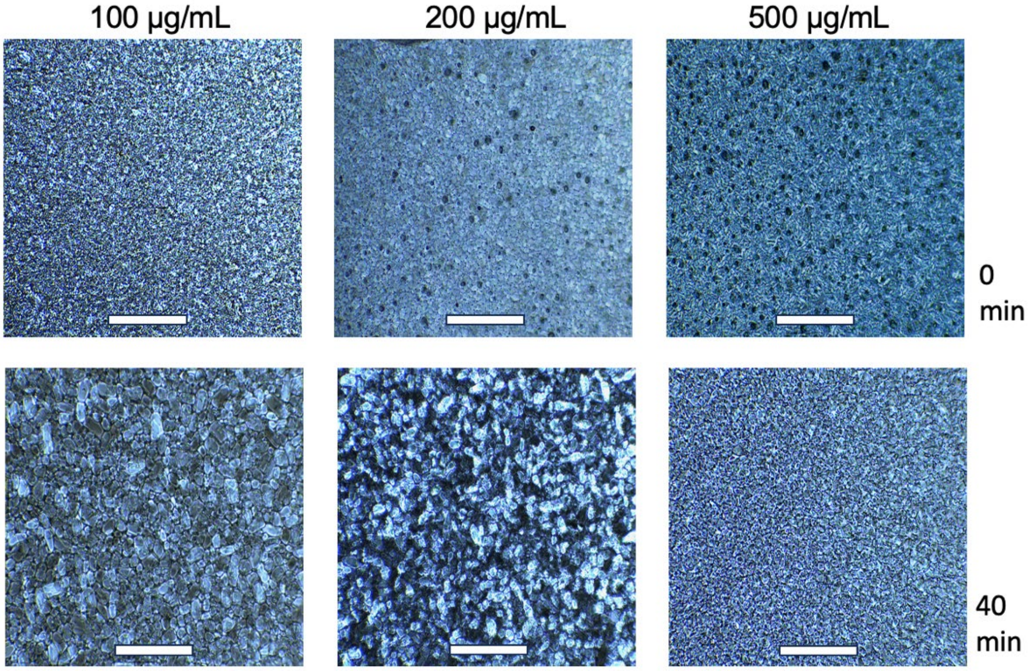

Figure S4. Ice recrystallization inhibition assay for Method B, hexylamine-initiated, $(A^LE^L)_{50}$ at varied concentrations in PBS at 0 minutes and 40 minutes of crystal growth. Scale bar is 200 μm.

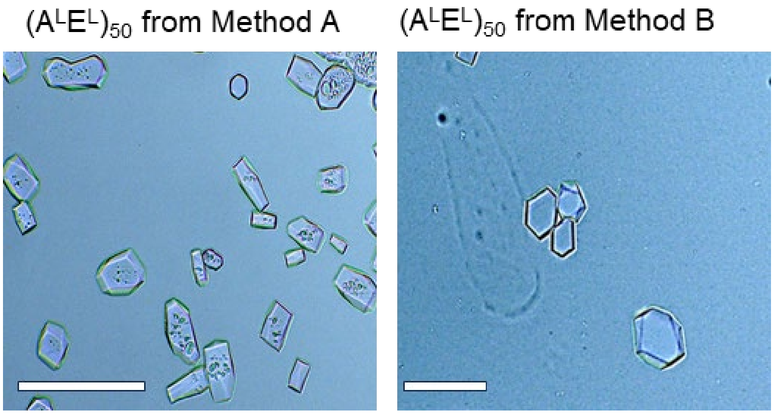

Figure S5. Dynamic ice shaping assay for $(A^LE^L)_{50}$ at 500 μg/mL mg/mL in PBS, derived from polymerization Method A or B. Structures bind and shape ice in an equivalent fashion. Scale bars are 100 μm.

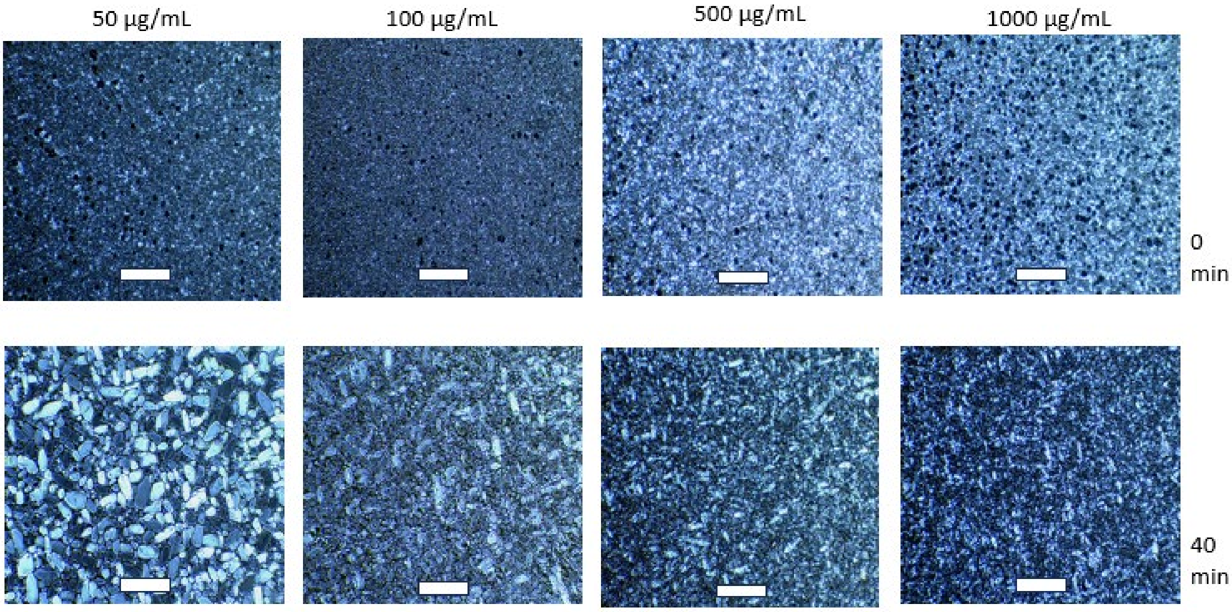

Figure S6. Ice recrystallization inhibition assay for $(A^DE^D)_{50}$ at varied concentrations in PBS at 0 minutes and 40 minutes of crystal growth. Scale bar is 200 µm.

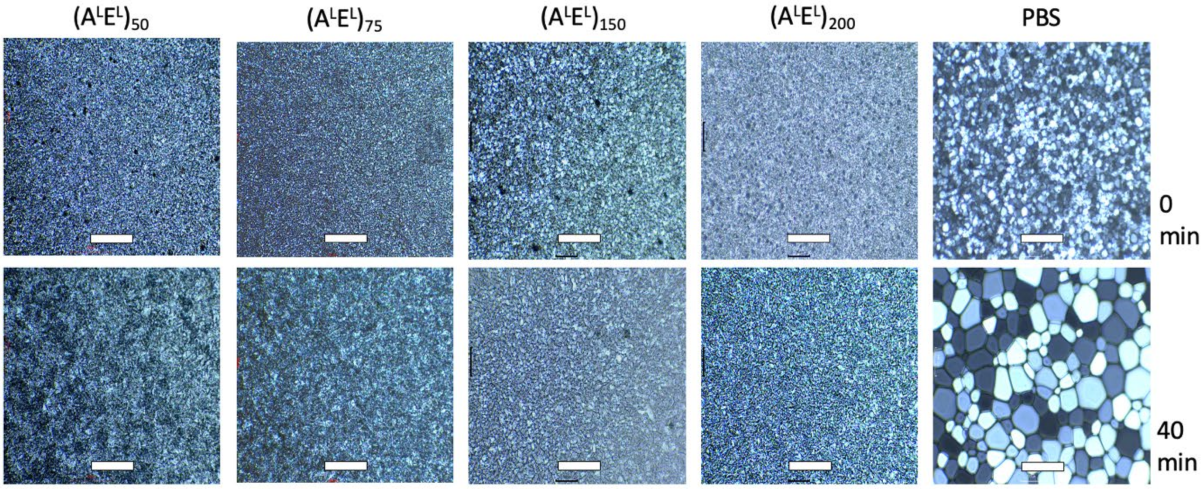

Figure S7. Ice recrystallization inhibition assay for $(A^LE^L)_n$ where images show crystal size at 0 minutes and 40 minutes of crystal growth. Polymer concentrations are 5 mg/mL in PBS. Scale bar is 200 µm.

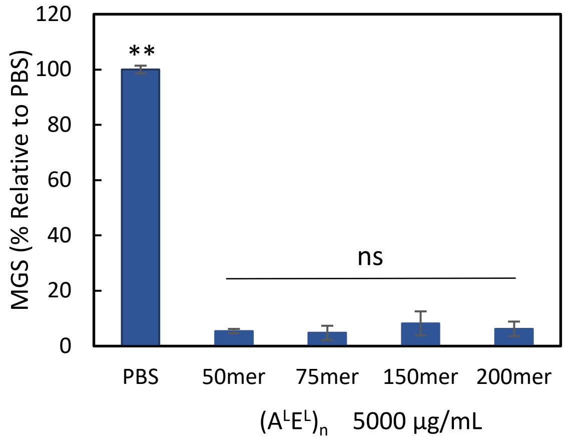

Figure S8. Quantified IRI of $(A^LE^L)_n$ at 5 mg/mL in PBS after 40 minutes of crystal growth. Standard deviation; ** indicates $p < 0.01$.

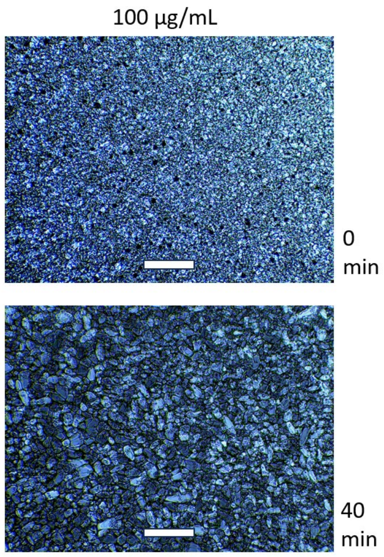

Figure S9. Ice recrystallization inhibition assay for $(A^LE^L)_{30}$ at 100 µg/mL in PBS at 0 minutes and 40 minutes of crystal growth. Scale bar is 200 µm.

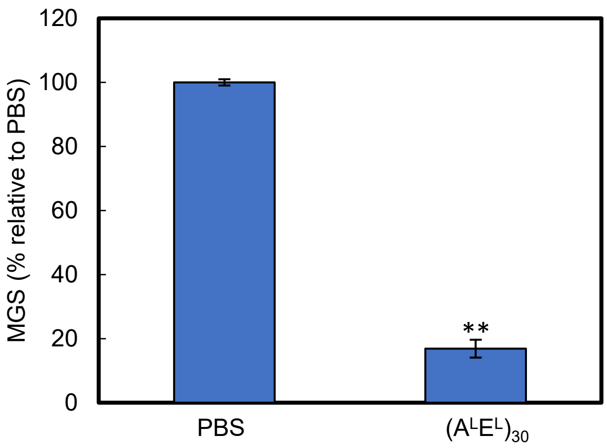

Figure S10. Quantified IRI of $(A^LE^L)_{30}$ at 100 μg/mL in PBS after 40 minutes of crystal growth. Standard deviation; ** indicates $p < 0.01$.

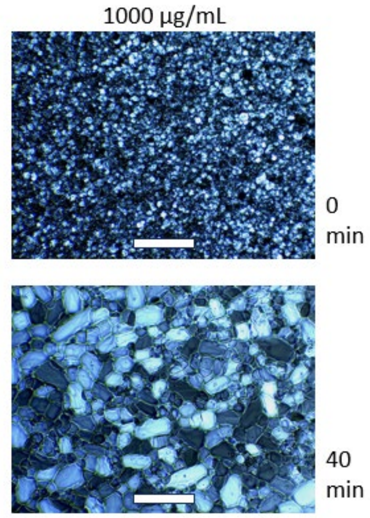

Figure S11. Ice recrystallization inhibition assay for $(V^LE^L)_{50}$ at 1000 μg/mL in PBS at 0 minutes and 40 minutes of crystal growth Scale bar is 200 μm.

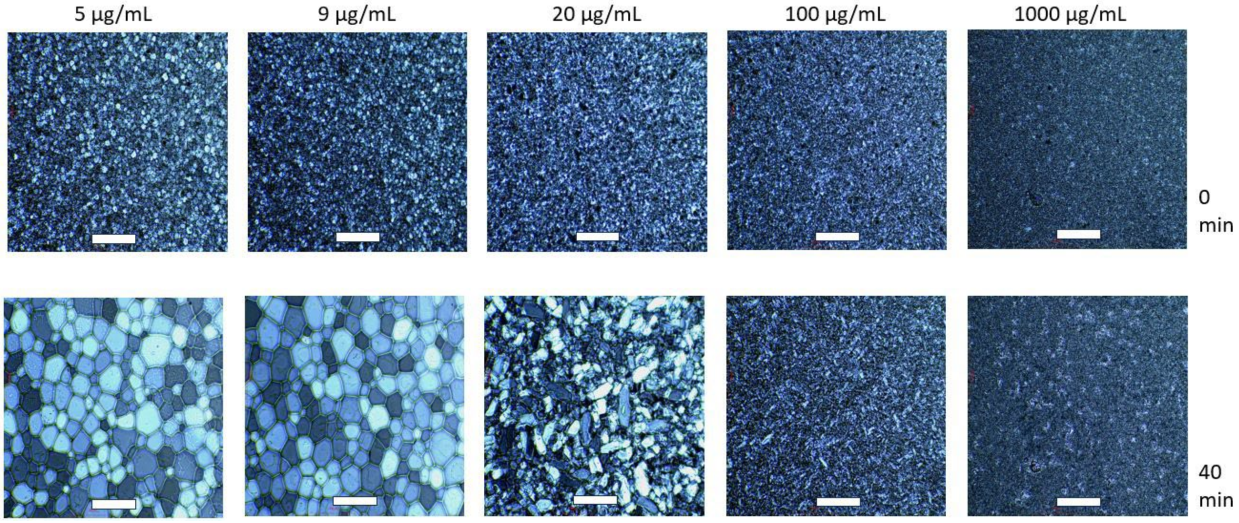

Figure S12. Ice recrystallization inhibition assay for $(A^{L}K^{L})_{50}$ at varying concentrations in PBS, after 0 minutes and 40 minutes of crystal growth. Scale bar is 200 µm.

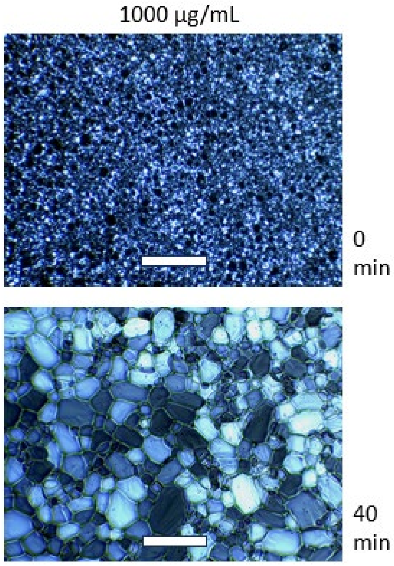

Figure S13. Ice recrystallization inhibition assay for racemic $(A^{D/L}E^{D/L})_{50}$ at 1000 µg/mL in PBS at 0 minutes and 40 minutes of crystal growth. Scale bar is 200 µm.

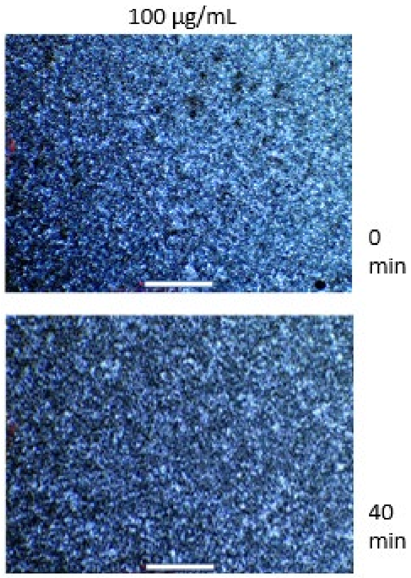

Figure S14. Ice recrystallization inhibition assay for wfAFP1 after heating to 95 °C for 10 minutes. Sample is 100 µg/mL in PBS and images are after 0 minutes and 40 minutes of crystal growth. Scale bar is 200 µm.

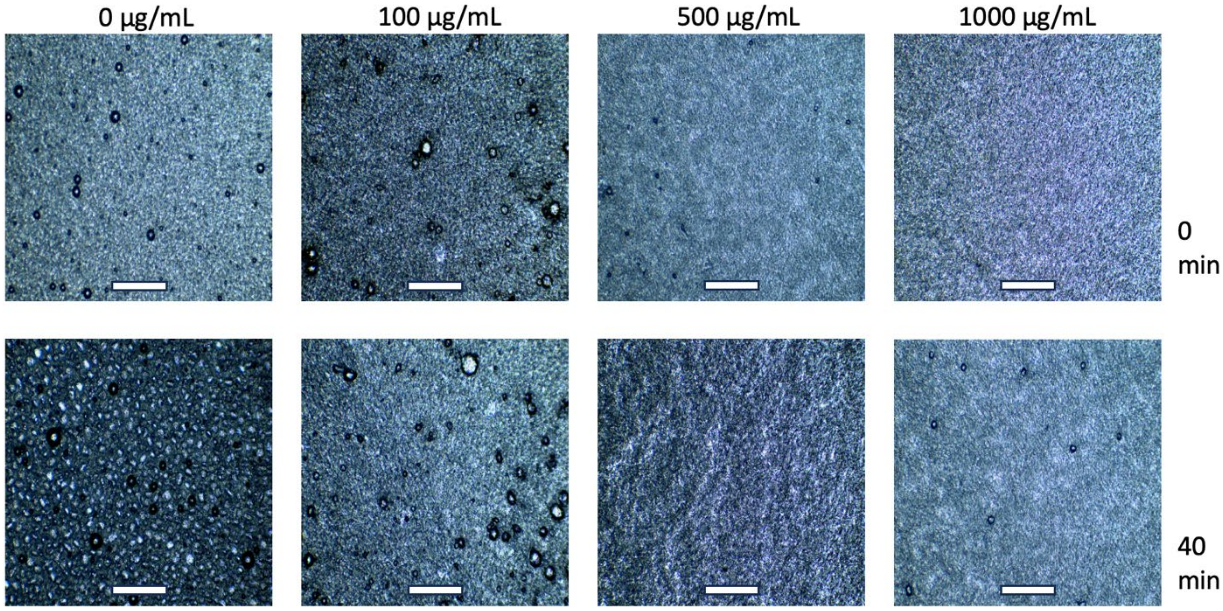

Figure S15. Ice recrystallization inhibition assay of frozen dairy product at 0 minutes and 40 minutes of crystal growth with and without the addition $(A^{L}E^{L})_{50}$. Scale bar is 200 µm.

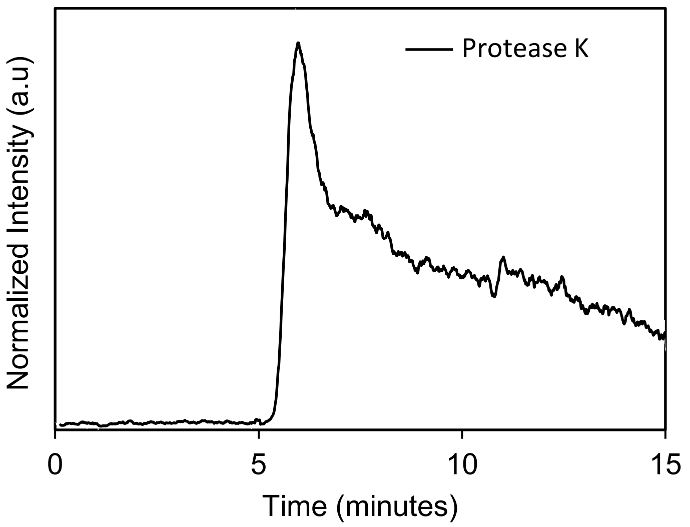

Fig S16. SEC/MALS trace in DPBS of Protease K.

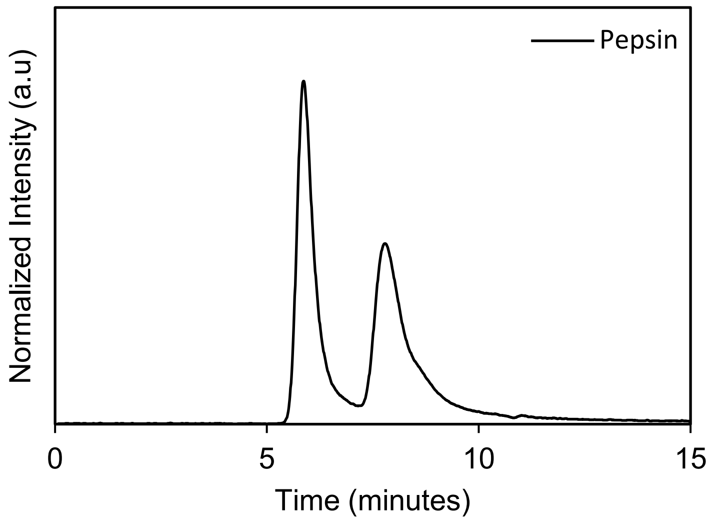

Fig S17. SEC/MALS trace in DPBS of Pepsin.

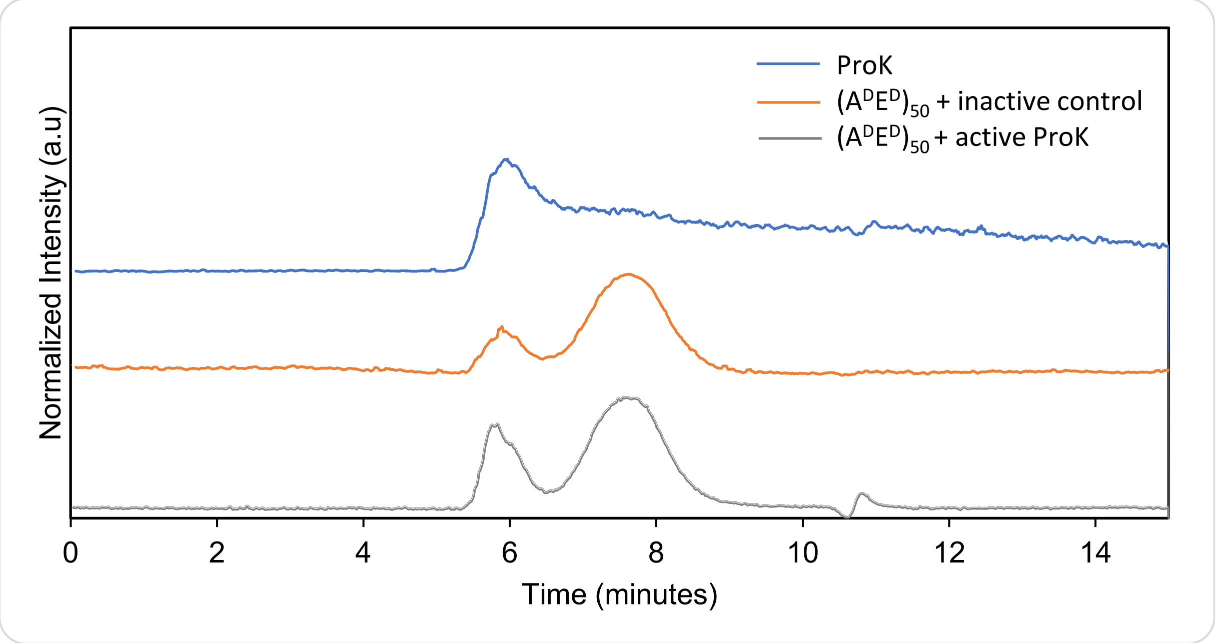

Figure S18. Stacked SEC/MALS traces in DPBS of ProK, control copolypeptide, and copolypeptide treated with active enzyme for 24 hrs at 37 °C.

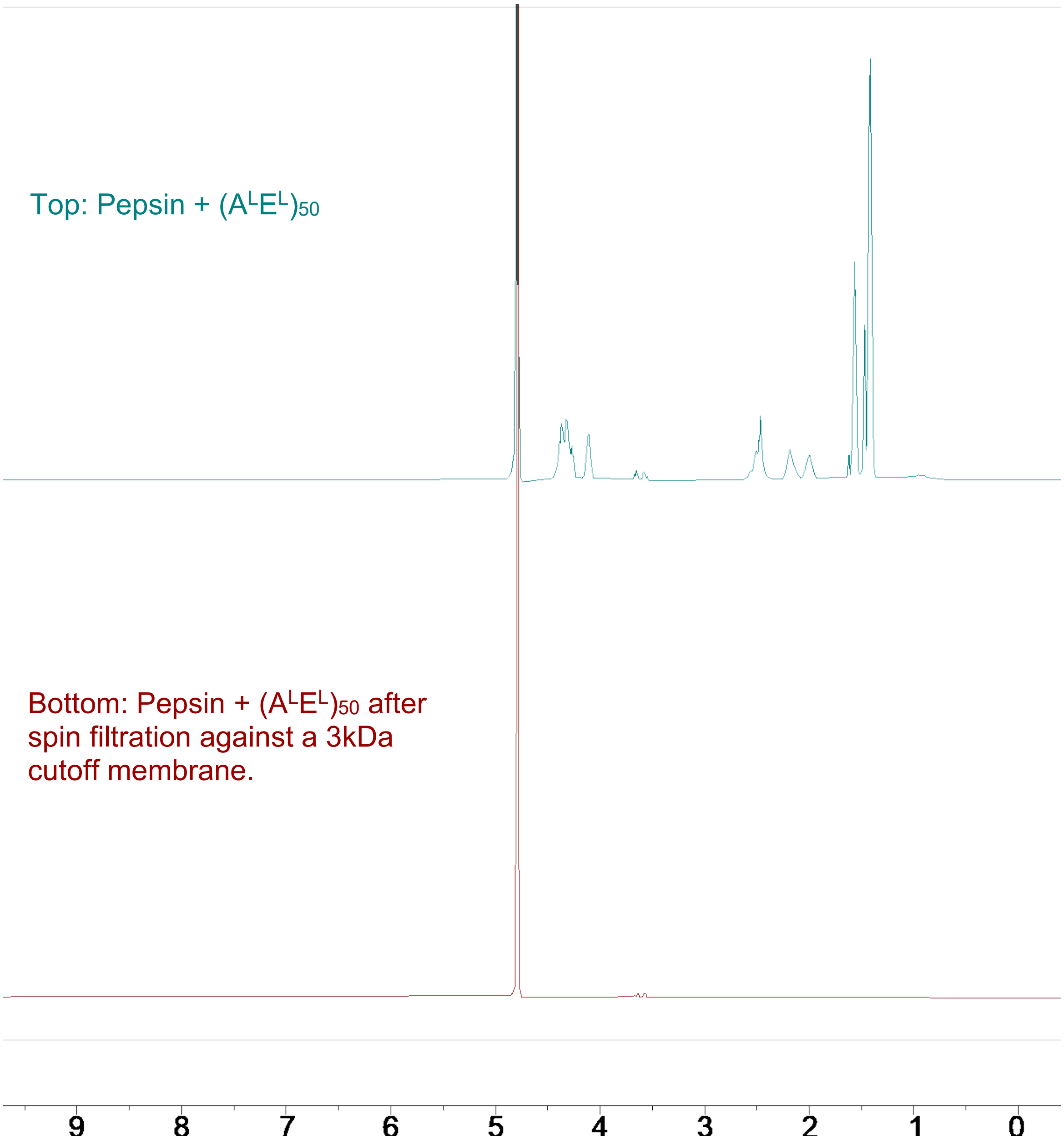

Figure S19: Pepsin enzymatic degradation experiment with $(A^{L}E^{L})_{50}$. Pepsin and $(A^{L}E^{L})_{50}$ were incubated in a 1:10 ratio for 24 hours and the reaction was analyzed by ^{1}H-NMR in D_2O. The top spectrum is the intact reaction, while the bottom spectrum is the reaction after spin filtration against a 3kDa membrane, indicating the enzyme has digested $(A^{L}E^{L})_{50}$ into fragments below this cutoff size.

III. NMR Spectra

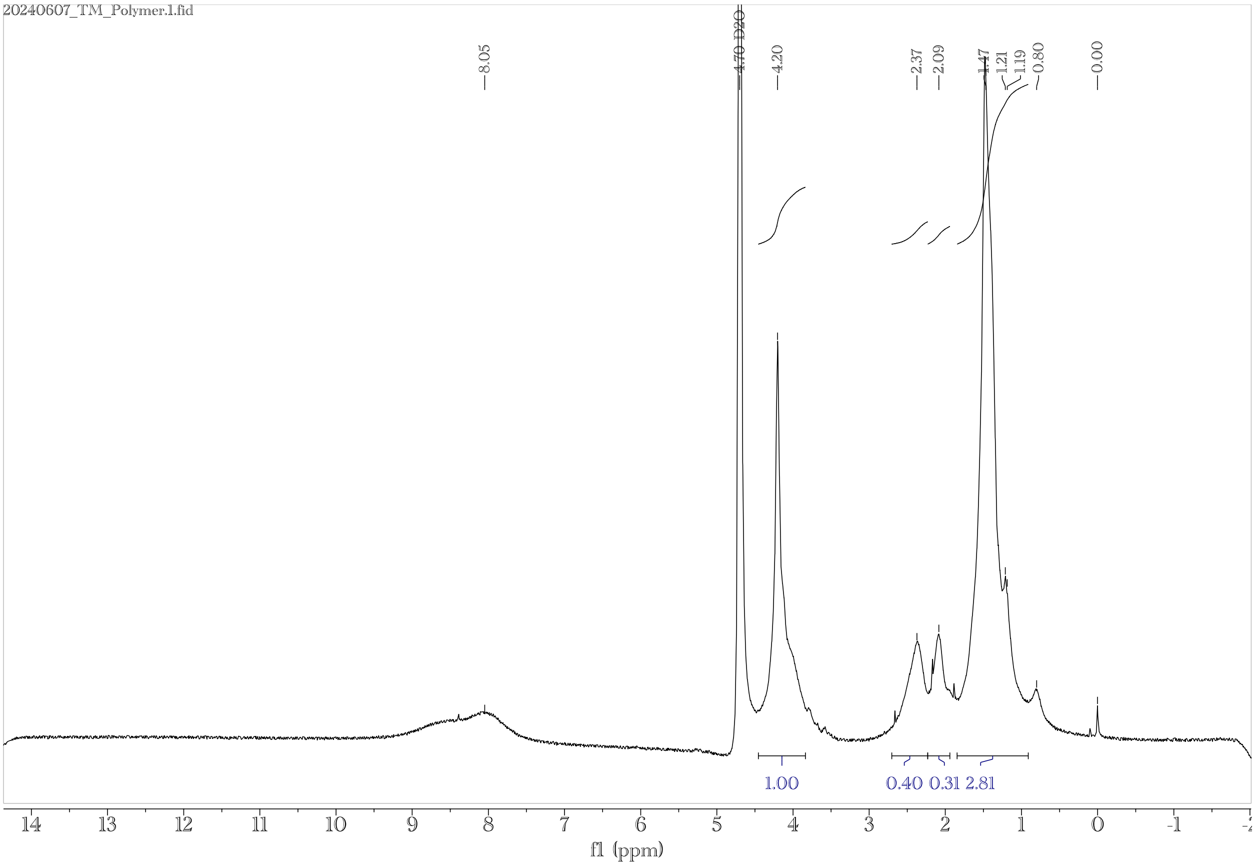

Figure S20. $(A^LE^L)_{50}$ ^{1}H-NMR in D_2O.

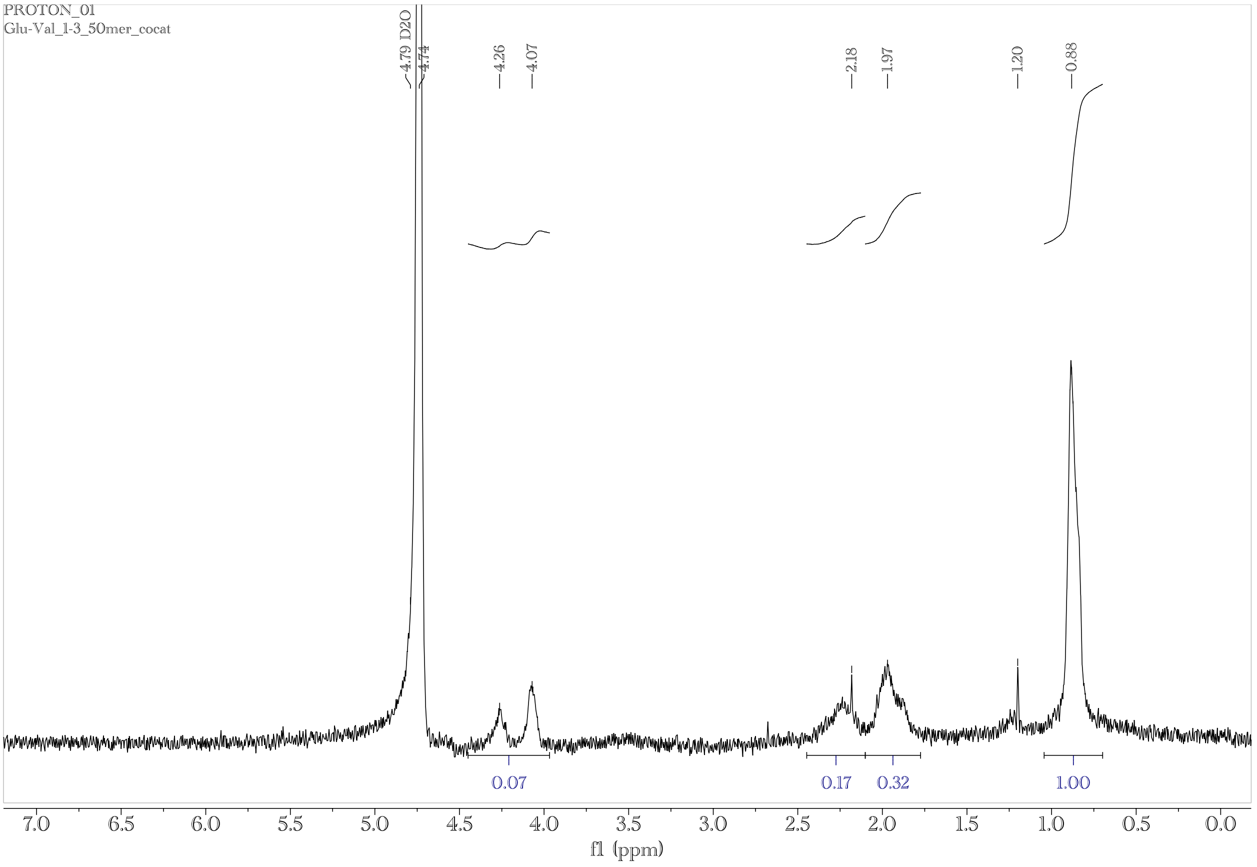

Figure S21. (V^LE^L)$_{50}$ ^{1}H-NMR in D_2O.

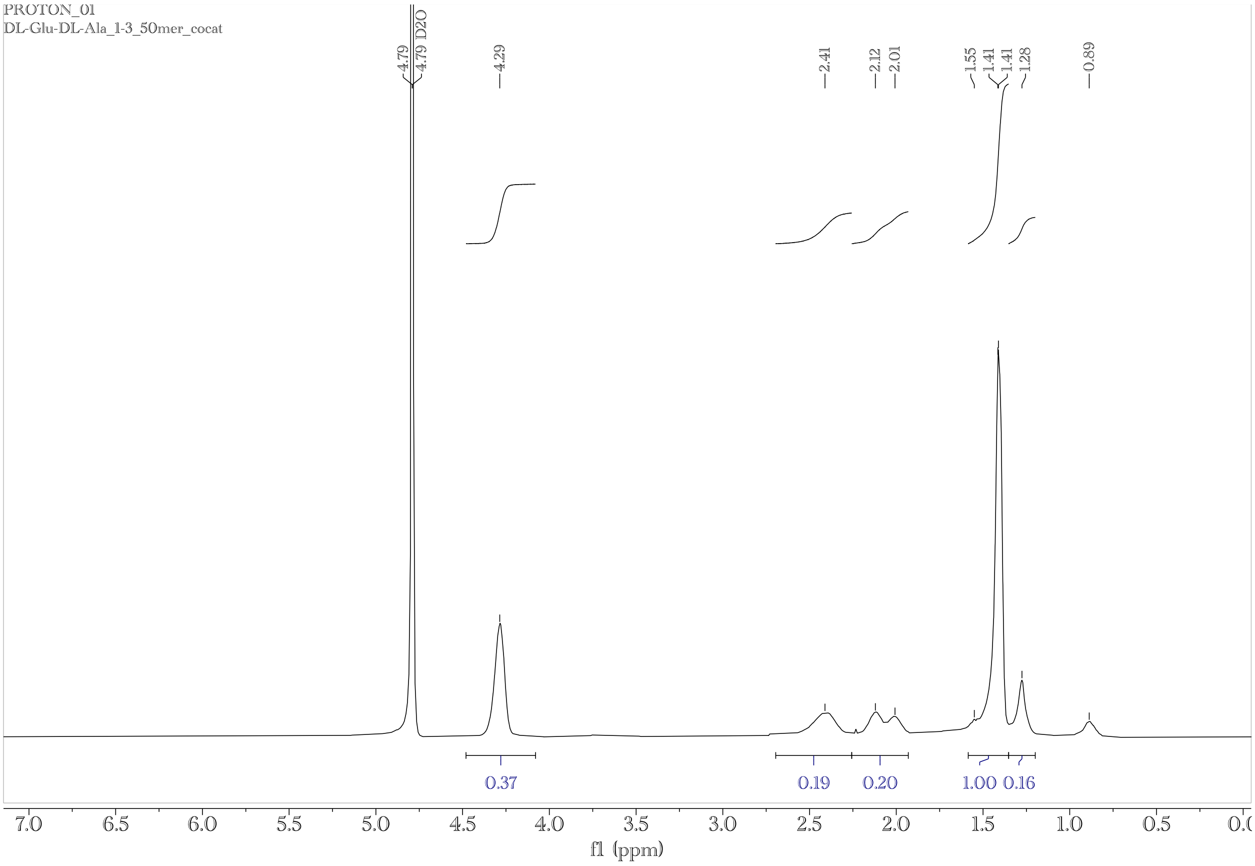

Figure S22. ($A^{D/L}E^{D/L}$)$_{50}$ ^{1}H-NMR in D_2O.

IV. ATR-FTIR

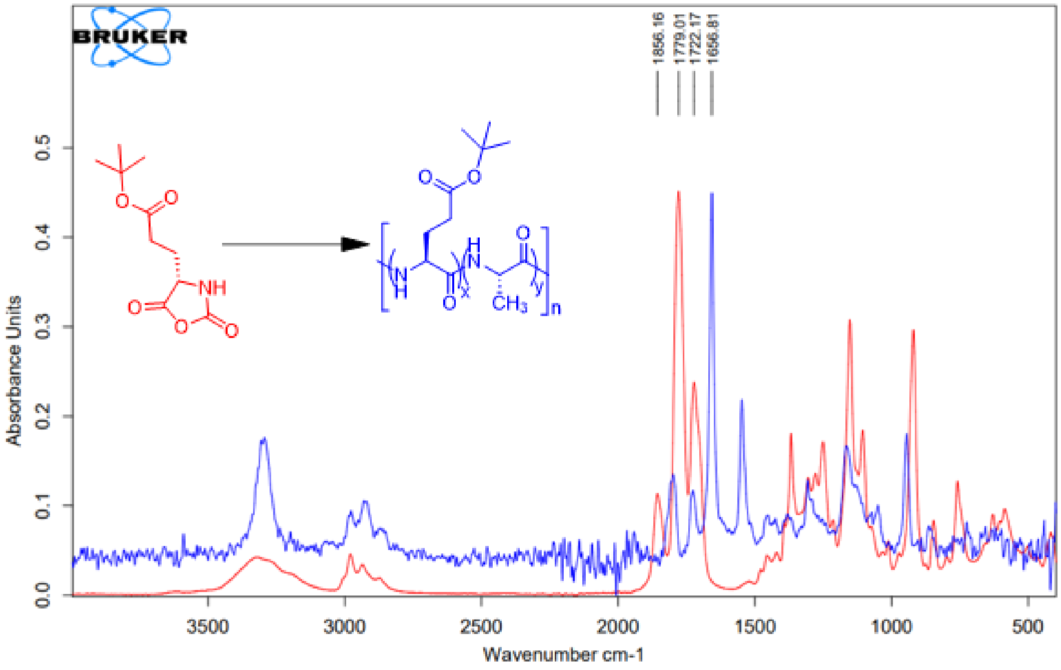

Figure S23. ATR-FTIR showing the disappearance of *t*Bu-E^{L} NCA (red) and the formation of (A^{L}*t*Bu-E^{L})$_{50}$ (blue).

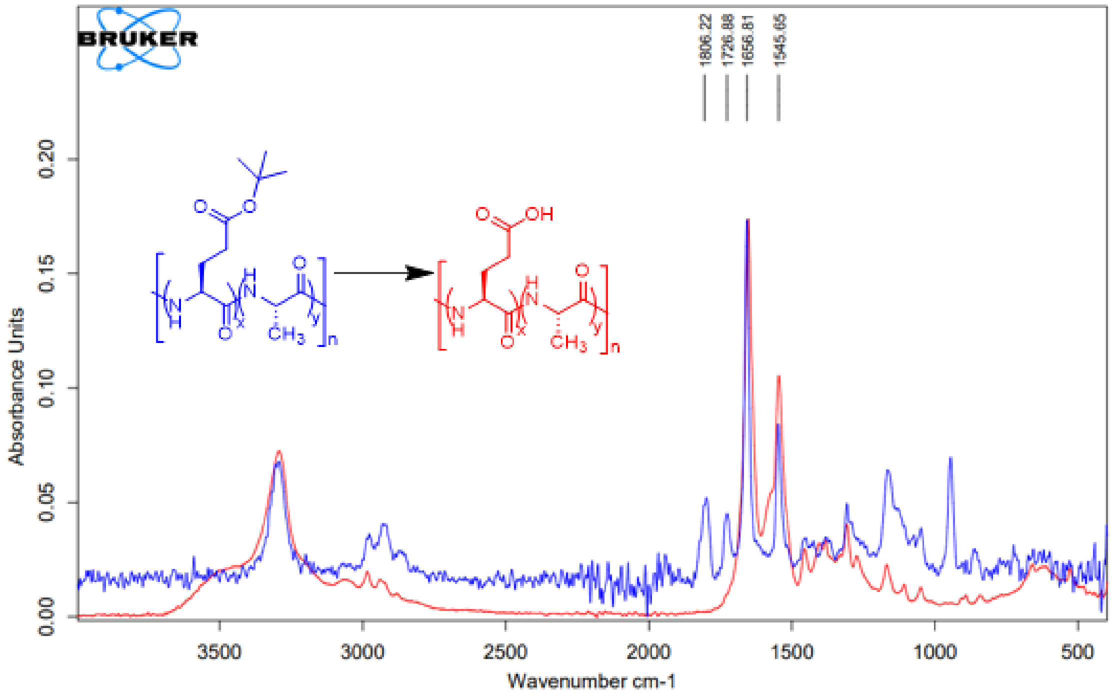

Figure S24. ATR-FTIR showing the disappearance of the *t*Bu group (A^{L}*t*Bu-E^{L})$_{50}$ (blue) and the formation of (A^{L}E^{L})$_{50}$ (red).

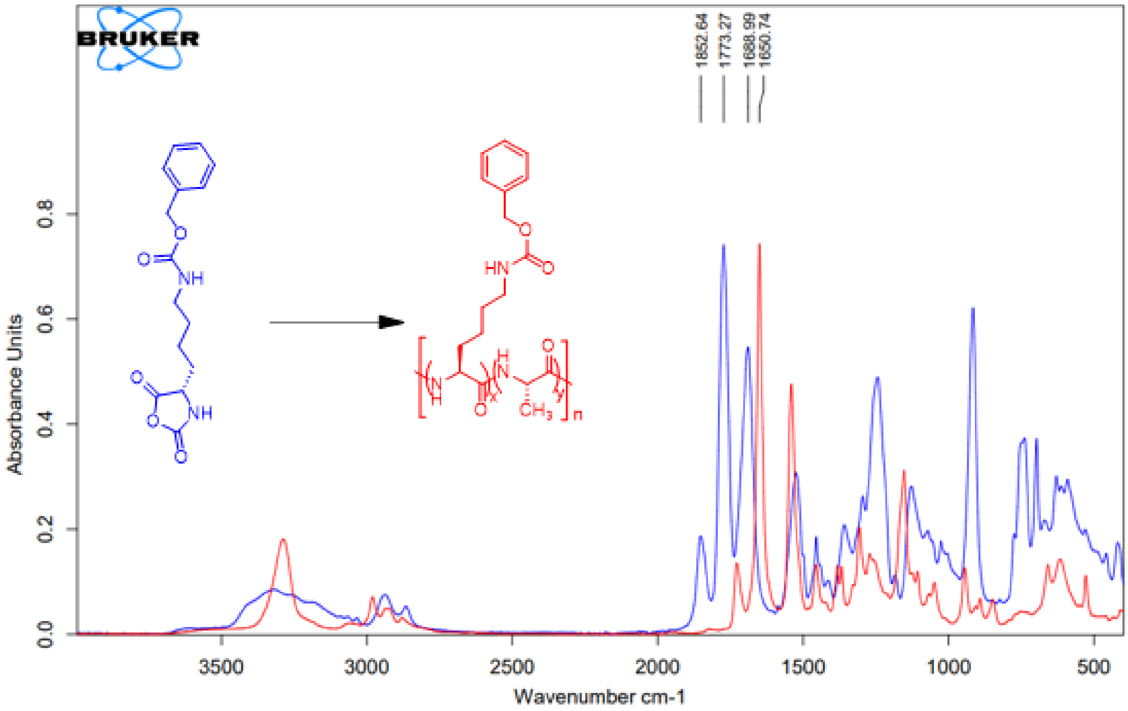

Figure S25. ATR-FTIR showing the disappearance of Z-K^{L} NCA (blue) and the formation of (A^{L}Z-K^{L})$_{50}$ (red).

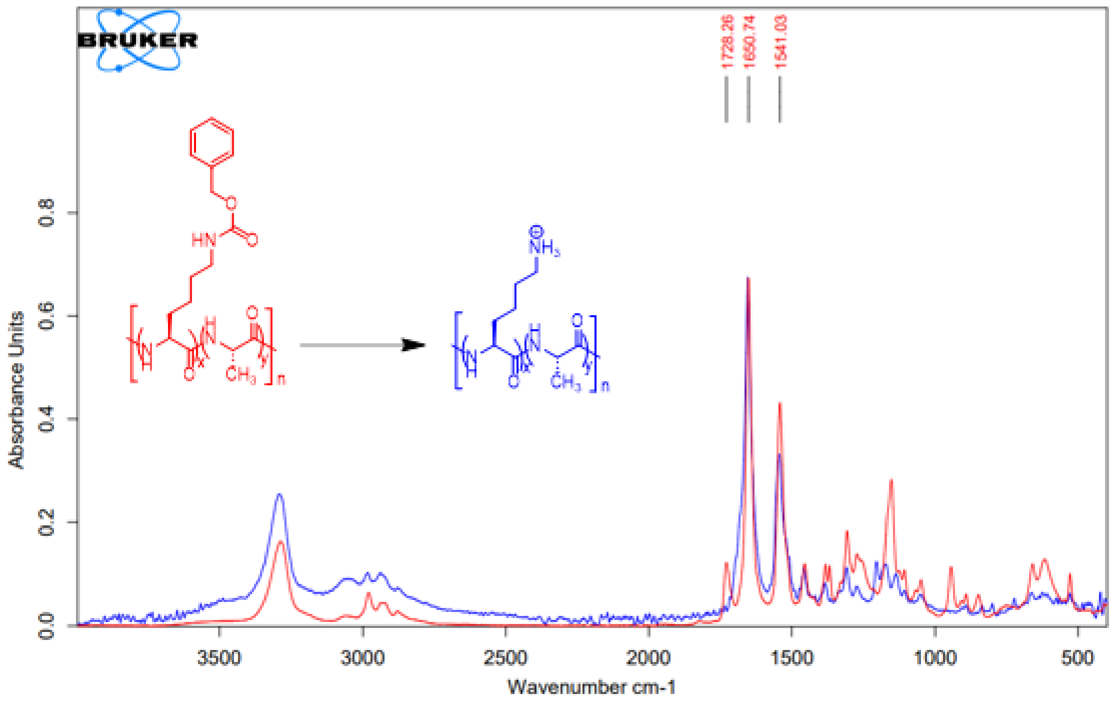

Figure S26. ATR-FTIR showing the disappearance of the Z protecting group from $(A^{L}Z\text{-}K^{L})_{50}$ (red) and the formation of $(A^{L}K^{L})_{50}$ (blue)
